# Platelet Lysate Activates Human Subcutaneous Adipose Tissue Cells by Promoting Cell Proliferation and Their Paracrine Activity Toward Epidermal Keratinocytes

**DOI:** 10.3389/fbioe.2018.00203

**Published:** 2018-12-21

**Authors:** Alessio Romaldini, Maddalena Mastrogiacomo, Ranieri Cancedda, Fiorella Descalzi

**Affiliations:** Department of Experimental Medicine (DIMES) and Department of Internal Medicine (DIMI), University of Genoa; Biotherapy Unit, IRCCS Ospedale Policlinico San Martino, Genoa, Italy

**Keywords:** regenerative medicine, mesenchymal stem cells, platelets, skin lesions, adipogenesis

## Abstract

Skin chronic wounds are non-healing ulcerative defects, which arise in association with a morbidity state, such as diabetes and vascular insufficiency or as the consequence of systemic factors including advanced age. Platelet Rich Plasma, a platelet-rich blood fraction, can significantly improve the healing of human skin chronic ulcers. Given that the subcutaneous adipose tissue is located beneath the skin and plays a role in the skin homeostasis, in this study, we investigated the *in vitro* response of human subcutaneous adipose tissue cells to platelet content in a model mimicking *in vitro* the *in situ* milieu of a deep skin injury. Considering that, at the wound site, plasma turn to serum, platelets are activated and inflammation occurs, human adipose-derived stromal cells (hASC) were cultured with Human Serum (HS) supplemented or not with Platelet Lysate (PL) and/or IL-1α. We observed that HS sustained hASC proliferation more efficiently than FBS and induced a spontaneous adipogenic differentiation in the cells. PL added to HS enhanced hASC proliferation, regardless the presence of IL-1α. In the presence of PL, hASC progressively lessened the adipogenic phenotype, possibly because the proliferation of less committed cells was induced. However, these cells resumed adipogenesis in permissive conditions. Accordingly, PL induced in quiescent cells activation of the proliferation-related pathways ERK, Akt, and STAT-3 and expression of Cyclin D1. Moreover, PL induced an early and transient increase of the pro-inflammatory response triggered by IL-1α, by inducing COX-2 expression and secretion of a large amount of PGE_2_, IL-6, and IL-8. Media conditioned by PL-stimulated hASC exerted a chemotactic activity on human keratinocytes and favored the healing of an *in vitro* scratch wound. In order to bridge the gap between *in vitro* results and possible *in vivo* events, the stimuli were also tested in *ex vivo* cultures of *in toto* human adipose tissue biopsies (hAT). PL induced cell proliferation in hAT and outgrowth of committed progenitor cells able to differentiate in permissive conditions. In conclusion, we report that the adipose tissue responds to the wound microenvironment by activating the proliferation of adipose tissue progenitor cells and promoting the release of factors favoring wound healing.

## Introduction

When a tissue or an organ is affected by an injury, the human body responds immediately by activating the wound-healing cascade. However, repair of large and deep lesions often results in fibrosis and scar formation, leading to a function reduction or a loss. Regenerative medicine was born and is developing with the aim to promote the regeneration of a fully functional tissue or organ structure and to prevent or delay the scarring process. “Classical” regenerative strategies are based on separated or combined use of biomaterials and stem/progenitor cells. However, due to regulatory, logistic and economical issues, they are still not broadly used in the clinical practice. It is our believe that the new frontier of regenerative medicine should head toward approaches able to reactivate and enhance dormant endogenous regenerative mechanisms (Cancedda et al., 2017). The fundamental condition for designing and developing these new clinically relevant tools is to know in detail the natural tissue response to an injury in order to identify the pathways to be activated and the ones to be limited.

In humans, the postnatal epidermis is one of the tissues with the highest regenerative potential. However, when the injury affects the underlying dermal layer, or it is repetitive, or when the wound healing process itself becomes defective, an aberrant repair process of skin lesions can take place determining serious consequences, such as a pathological scarring (hypertrophic scar or keloid) or a chronic wound (Wynn and Ramalingam, 2012; Eming et al., 2014). In particular, chronic wounds are non-healing ulcerative skin defects, which arise in association with a morbidity state (e.g., vascular diseases or diabetes mellitus) or in the presence of systemic factors (e.g., advanced age), and represent a very important worldwide medical issue and economic burden. In developed countries, it has been estimated that 1–2% of the population will experience a chronic ulcer during their lifetime (Sen et al., 2009). For the treatment of chronic ulcers, new therapeutic approaches are based on *in situ* administration of bioactive molecules, incorporated or not in wound dressings, to enhance the physiological healing process (Eming et al., 2014). In this scenario, it was reported that Platelet Rich Plasma (PRP), a platelet-rich blood fraction, could improve the healing of chronic wounds (Martinez-Zapata et al., 2012; Sarvajnamurthy et al., 2013; Suthar et al., 2017).

Other platelet by-products were also described, such as platelet-released supernatant obtained by the activation of platelets (Kandler et al., 2004; Giusti et al., 2013) or platelet lysate (PL) obtained by the lysis of platelets (Barsotti et al., 2013; Ruggiu et al., 2013; Antoninus et al., 2015), and tested in several cell systems. By focusing our attention on the molecular mechanisms activated by platelet-derived factors in skin wound healing, we previously investigated the effects of PL on human keratinocytes and we showed that PL-stimulated resting cells transiently produced increased levels of the inflammatory cytokine IL-8 and of the antimicrobial peptide NGAL, via p38 MAPK and NF-κB activation, and that the wound closure in an *in vitro* scratch assay was accelerated upon PL stimulation (El Backly et al., 2011).

In this study, we addressed the possible role played by the human subcutaneous adipose tissue in supporting the repair/regeneration process of skin wounds, given that such tissue is located beneath the skin and it physiologically contributes to re-establish the homeostasis of the damaged skin. More specifically, we investigated the *in vitro* response of the human subcutaneous adipose tissue and derived cells to the platelet content. For this purpose, we defined a clinically relevant model using the early injury-associated stimuli, which are Human Serum (HS), Platelet Lysate (PL) and Interleukin-1α (IL-1α), in order to reproduce *in vitro*, as much as possible, the *in situ* microenvironment established following a deep skin injury. In particular, HS played the role of the plasma-derived serum, which is the physiological fluid at the wound site, while PL corresponded to that well-balanced cocktail of bioactive molecules released by platelets and involved in all steps of the wound healing process (Golebiewska and Poole, 2015; Cancedda et al., 2017). Because of the adopted manufacturing procedures, HS was not contaminated by platelet-derived factors and PL did not contain plasmatic molecules, thus allowing studies on their separated and combined effects on adipose-tissue cells. The IL-1α is physiologically involved in the induction and the maintenance of local inflammatory responses (Di Paolo and Shayakhmetov, 2016) and it was used to mimic the inflammatory milieu of the wound. The reported model allowed to dissect the system and to specifically elucidate the effects of platelet content on subcutaneous adipose tissue in order to give a rationale to the therapeutic use of PL in chronic wounds. The considered stimuli were tested in both primary cultures of human adipose-derived stromal cells (hASC), used as *in vitro* paradigm of resident progenitor cells, and in *ex vivo* cultures of *in toto* human adipose tissue biopsies (hAT). We studied the PL effect on HS-expanded cell proliferation, both in the presence and in the absence of IL-1α, and on cell differentiation upon short or long PL exposure. We also evaluated the effects of media conditioned by PL-stimulated cells on keratinocyte migration by an *in vitro* scratch assay. We, finally, studied PL effects on hAT cultured *ex vivo* in order to bridge the gap between *in vitro* results and *in vivo* events.

## Materials and Methods

### Materials

Type I collagenase was purchased from Worthington (Lakewood, NJ, USA). The MEM Alpha Medium (1X) GlutaMAX™ without nucleosides (αMEM) was from Gibco (Thermo Fisher Scientific, Waltham, MA, USA). Minimum essential medium (MEM) with Earle's Salts, fetal bovine serum (FBS), penicillin G-streptomycin sulfate solution, L-glutamine and normal goat serum were from Euroclone Life Sciences Division (Milan, Italy). Dimethyl sulphoxide (DMSO) and Harris hematoxylin were obtained from PanReac AppliChem (Barcelona, Spain). Human interleukin-1alpha (IL-1α) was from PeproTech (London, UK). Bright-Line™ Hemacytometer with an improved Neubauer chamber, crystal violet powder, thiazolyl blue tetrazolium bromide (MTT), insulin from bovine pancreas, dexamethasone, indomethacin, 3-isobutyl-1-methylxanthine (IBMX), L(+)-ascorbic acid sodium salt, β-glycerophosphate disodium salt hydrate, Oil Red O (ORO), Alizarin Red S (ARS), paraformaldehyde, protease inhibitor cocktail and poly-L-lysine solution were purchased from Sigma-Aldrich (St. Louis, MO, USA). Formaldehyde 37% weight solution was from Chem-Lab (Zedelgem, Belgium). Methylene blue hydrate powder was purchased from Honeywell Riedel-de Haën® (Seelze, Germany). The RNeasy® Plus Micro Kit and Omniscript® RT Kit were from Qiagen (Milan, Italy). Power SYBR® Green PCR Master Mix and 7500 Fast Real-Time PCR System were from Applied Biosystems® Life Technologies (Thermo Fisher Scientific, Waltham, MA, USA). The NuPAGE™ 4–12% Bis-Tris gels and streptavidin-peroxidase of HistoMouse™-SP kit were from Invitrogen (Thermo Fisher Scientific, Waltham, MA, USA). Amersham™ Protran™ 0.45 μm NC, secondary anti-rabbit and anti-mouse horseradish peroxidase-linked immunoglobulins G (IgGs), Amersham™ ECL™ western blotting detection reagents and Amersham™ Hyperfilm™ ECL were obtained from GE Healthcare (Buckinghamshire, UK). Primary antibodies raised against interleukin-8 (IL-8), interleukin-6 (IL-6), Cyclin D1 and Actin were purchased from Santa Cruz Biotechnology Inc. (Dallas, TX, USA). Primary antibodies raised against phospho-Akt, Akt, phospho-ERK_1/2_, ERK_1/2_, phospho-STAT3, and STAT3 were acquired from Cell Signaling Technology (Danvers, MA, USA). Primary antibody raised against cyclooxygenase-2 (COX-2) and Prostaglandin E_2_ ELISA kit were from Cayman Chemical (Ann Arbor, MI, USA). Secondary anti-goat peroxidase-conjugated immunoglobulins G (IgGs) were from Jackson ImmunoResearch (Bar Harbor, MA, USA). Eosin Y was obtained from Carlo Erba Reagents (Milan, Italy). Eukitt® was purchased from O. Kindler GmbH & Co (Freiburg, Germany). Primary antibody raised against PCNA was from Abnova (Taipei City, Taiwan). Secondary biotinylated immunoglobulins G (IgGs) and 3,3′-diaminobenzidine (DAB) were from Dako (Carpinteria, CA, USA). Superfrost Ultra Plus Slides were obtained from Fisher Scientific (Thermo Fisher Scientific, Waltham, MA, USA).

### HS and PL Production

The Human Serum (HS) and the Platelet Lysate (PL) were derived from expired blood donated to the Blood Transfusion Center of IRCCS Ospedale Policlinico San Martino (Genova, Italy). At the time of blood donation, all donors provided the written informed consent for the use of donated blood for scientific applications. Both reagents were produced from pool of anonymized blood donations (at least 3 donors for HS or 25 donors for PL) in order to minimize variations among donors. The plasma and the buffy coat containing platelets, red blood cells and other particulate blood components were separated by standard procedures of the Blood Transfusion Center. The plasma was frozen and thawed at 4°C and the produced cryoprecipitate containing fibrinogen and other coagulation factors was removed. A complete clotting was obtained by adding calcium chloride (2 mg/mL) at 37°C for 6 h. After a high-speed centrifugation, the supernatant (i.e., HS, devoid of platelet content) was collected, divided into aliquots, lyophilized and stored at −20°C until use.

For the PL production, the buffy coats were centrifuged at low speed and the lower phase, enriched in platelets (platelet-rich plasma, PRP), was separated, pooled, and centrifuged at high speed to sediment the platelets. The platelets were washed 3 times with physiological saline (0.9% w/v NaCl) by re-suspension of the pellet in the physiological saline and sedimentation by centrifugation, in order to eliminate possible plasma-derived contaminant. The final pellet was re-suspended at a concentration of 10 × 10^6^ platelets/μL and subjected to 3 consecutive freeze/thaw cycles. After a high-speed centrifugation, the supernatant containing the cocktail of platelet-released factors (i.e., PL) was collected, divided into aliquots, lyophilized, and stored at −20°C. PL was added to culture medium at a concentration of 5% (v/v), approximately corresponding to the highest physiological concentration of platelets in the human blood, without any addition of heparin.

### Primary hASC Cultures

Primary hASC were obtained from anonymized subcutaneous adipose tissue liposuction wastes from healthy subjects. All donors provided the written informed consent. Protocol and procedures were approved by the Local Ethical Committee. The hASC were isolated following the protocol reported by Estes et al. (2010), with some modifications. Briefly, the liposuction aspirate was combined with phosphate-buffered saline (PBS) pH 7.4 (1:1 volume), shacked, and let stand to allow the separation of the aqueous (lower) and the fat (upper) phases. The fat phase was collected, combined with fresh PBS and the procedure repeated several times until the aqueous phase appeared clear. The fat phase was then digested with 0.1% type I collagenase in PBS (1:1 volume) at 37°C for 1 h. The sample was centrifuged at 290 g for 5 min at room temperature and the pellet was re-suspended in the supernatant by shacking vigorously the tube in order to ensure that all stromal cells were properly released and separated from the remaining tissue. After a further centrifugation at 290 g, the upper mature adipocyte and the middle collagenase/PBS layers were aspirated off leaving the pellet, which contained the stromal vascular fraction (SVF). The pellet was re-suspended in αMEM supplemented with 10% (v/v) FBS to neutralize residual collagenase, centrifuged, re-suspended in PBS and split in two half. After a final centrifugation, one half was re-suspended in αMEM, the most appropriate basal medium for long-term culture of hASC (Dhanasekaran et al., 2012), supplemented with 10% (v/v) HS, 100 U/mL penicillin G and 100 μg/mL streptomycin sulfate, while the other half was re-suspended with an identical volume of αMEM supplemented with 10% (v/v) FBS, 100 U/mL penicillin G and 100 μg/mL streptomycin sulfate. Cells were plated at an equivalent to about 0.04 mL of liposuction aspirate per cm^2^, incubated at 37°C in a humidified atmosphere with 5% CO_2_ and media were changed 3 times per week. At 80% confluence, cells were harvested by trypsinization and frozen in liquid nitrogen using αMEM supplemented with 50% (v/v) HS or FBS, 10% (v/v) DMSO, 100 U/mL penicillin G and 100 μg/mL streptomycin sulfate for long-term storage (P0). After thawing, cells were expanded for one passage and, unless differently specified, used at P2; a cell viability of 90% to 100% was observed by trypan blue exclusion test. In some experiments HS-isolated hASC were cultured and expanded in HS containing medium supplemented with 5%PL.

### *In toto* hAT Cultures

*In toto* samples of human adipose tissue (hAT) were obtained from resection material of abdominoplasty on healthy females. All donors provided the written informed consent. The samples were anonymized. The inner portion of subcutaneous fat was minced into pieces with the diameter of 3–4 mm, paying attention to avoid visible blood vessels. Such samples were cultured in 24-well plates using complete culture medium supplemented with 5% PL, 100 U/mL IL-1α, or 5% PL + 100 U/mL IL-1α, or without any supplement, for 14 days. *In toto* hAT cultures were incubated at 37°C in a humidified atmosphere with 5% CO_2_ and media were changed 3 times per week.

### Human Keratinocyte Cultures

Human keratinocyte NCTC 2,544 cell line was obtained from “Centro di Risorse Biologiche” (CRB) of IRCCS Ospedale Policlinico San Martino (Genova, Italy). Keratinocytes were cultured following CRB's instructions, i.e., using MEM with Earle's Salts supplemented with 10% FBS, 2 mM L-glutamine, 100 U/mL penicillin G, and 100 μg/mL streptomycin sulfate. Keratinocyte cultures were incubated at 37°C in a humidified atmosphere with 5% CO_2_ and media were changed 3 times per week.

### Proliferation Assays

Cell proliferation was evaluated by two different procedures:

- Cell counting: HS- or FBS-isolated hASC at P2 were plated at 4.0 × 10^3^ cells/cm^2^/well in 6-well plates in the same medium adopted for their isolation. Cells were incubated for 24 h to enable their adhesion to wells and, the following day (day 0), the medium was replaced, according to the experiment performed, with: (i) FBS-containing control medium; (ii) HS-containing medium (complete medium); (iii) HS containing medium supplemented with 5% PL. For each culture condition, the cell number was measured in two different wells, after 0, 2, 4, 6, and 8 days of stimulation, using a Bright-Line™ Hemacytometer with an improved Neubauer chamber. The final result is expressed as the average of 3 independent experiments performed on different single-donor primary hASC cultures ± SD value.
- Crystal violet staining assay: HS-isolated hASC were plated at 2.5 × 10^3^ cells/well in 96-well plate and incubated in complete medium for 24 h. The next day (day 0), medium was replaced with complete HS-containing medium un-supplemented or supplemented with: (i) 5% PL; (ii) 100 U/mL IL-1α; (iii) 5% PL + 100 U/mL IL-1α. Crystal violet staining was performed in quintuplicate for each culture condition after 0, 1, 2, 3, and 4 days of stimulation, as described by Nguyen et al. (2017). For each culture condition, the final result is the n-fold increase of OD with respect to day 0, expressed as the average of 3 independent experiments performed on different single-donor primary hASC cultures ± SD value.

### Viability Assay

The hASC viability was evaluated in a parallel set of experiments by colorimetric MTT assay. In particular, HS- or FBS-isolated hASC at P2 were plated at 4 × 10^3^ cells/cm^2^/well in 24-well plates and, after an incubation of 24 h, media were changed and a growth curve was performed. In some experiments, 5% PL was added to the HS-containing medium and a growth curve determined. After 0, 2, 4, 6, and 8 days of stimulation, the assay was performed as reported by Muraglia et al. (2015). For each culture condition, the OD was measured in three different wells and the final results are the n-fold increase of OD with respect to first time-point values, expressed as the average of 3 independent experiments performed on different single-donor primary hASC cultures ± SD value.

### Colony-Forming Efficiency (CFE) Assay

Immediately after their isolation (P0), hASC were plated at 100 μL of lipoaspirate/Petri dish in HS-containing medium, un-supplemented or supplemented with 5% PL, or in FBS-containing control medium in duplicate per each culture condition. After 9 days, colonies were fixed by 10% neutral buffered formalin (10 mL formaldehyde 37% weight solution, 90 mL PBS) for 10 min at room temperature and stained with 1% methylene blue solution at pH 8.8 (1% w/v methylene blue hydrate powder, 0.2 M boric acid, 0.05 M sodium tetraborate decahydrate, distilled water up to the final volume) for 45 min at room temperature. In each dish, the colony number was counted by naked eye. The CFE was also quantified by using the ImageJ-plugin “ColonyArea,” which calculates two output parameters: “colony area percentage,” i.e., the percentage of dish area covered by colonies, and “colony intensity percentage” that takes into account both the area covered by colonies and the density of the colonies (Guzmán et al., 2014). The number of colonies and both the ColonyArea-derived parameters are expressed as mean values ± SD calculated on the basis of 5 independent experiments relative to different single-donor primary cultures for all tested culture conditions.

### Adipogenic and Osteogenic Differentiation

To induce adipogenic and osteogenic differentiation, hASC were plated at 8 × 10^3^ cells/cm^2^/well in 24-well plates and maintained in culture in complete medium until confluence. At confluence, one half of wells was cultured for 14 days with an induction medium specific for adipogenic or osteogenic differentiation, while the other half was cultured with complete medium as internal control. Adipogenic differentiation medium comprised either the complete medium supplemented with 6 ng/mL insulin and 10^−7^ M dexamethasone (standard differentiation medium) or the standard differentiation medium further supplemented with 200 μM indomethacin and 500 μM IBMX (Scott et al., 2011). Osteogenic differentiation medium was composed of complete medium supplemented with 50 μg/mL ascorbic acid, 10 mM β-glycerophosphate and 10^−7^ M dexamethasone (Muraglia et al., 2017). Media were changed 3 times per week. The presence of intracellular lipid droplets was revealed by Oil Red O (ORO) staining. To quantify incorporated ORO, ORO extraction was performed by incubating stained cell layers in 100% propan-2-ol for 10 min at room temperature with mild agitation and the recovered solution OD at 530 nm was determined. The assays were performed in triplicate and the results are expressed as the average of 3 independent experiments performed on different single-donor primary hASC cultures ± SD value. In the osteogenic differentiation experiments, matrix mineralization was evaluated by Alizarin Red S (ARS) staining. Osteogenic differentiation was tested in duplicate on 3 different single-donor primary cultures.

### Real Time Quantitative PCR

Total RNA was extracted by RNeasy® Plus Micro Kit following manufacturer's instructions from hASC cultured in different conditions and for different times, as described above. RNA concentrations were measured at 260 nm and RNA purity was checked considering 260 nm/280 nm ratio with values included in 1.5–2.1 range. Total RNA was reverse-transcribed to cDNA using Omniscript® RT Kit according to manufacturer's protocol. Transcript levels of target genes were measured by Real Time quantitative PCR using Power SYBR® Green PCR Master Mix on 7500 Fast Real-Time PCR System. Primer sequences were: forward 5′-CCTATTGACCCAGAAAGCGA-3′ and reverse 5′-GGGAGTGGTCTTCCATTACG-3′ for *ppar*γ*2* (designed by Primer-BLAST, Ye et al., 2012); forward 5′-CAGAGGGACCGGAGTTATGA-3′ and reverse 5′-TTCACATTGCACAAGGCACT-3′ for *c/ebp*α (Russo et al., 2014); forward 5′-GACTCAACACGGGAAACCTCACC-3′ and reverse 5′-ACCAGACAAATCGCTCCACCAACT-3′ for 18S rRNA (Schiller et al., 2013). The expression of 18S rRNA was used as endogenous control (normalizer). For each primer pair, the production of a single amplicon was checked by generating a melting curve. Transcript levels were extrapolated from a standard curve, generated using a dilution series of cDNA sample mixture, and divided by normalizer quantities. Each sample was assayed in triplicate. The final results are expressed as the average of 3 independent experiments performed on different single-donor primary hASC cultures ± SD values.

### Western Blot

To analyze the cytokine production, HS-isolated hASC at P2 were seeded at 1.0 × 10^5^ cells/dish in 60 mm dishes, cultured in complete medium until 80% confluence and treated for 24 h with serum-free medium supplemented with: (i) 5% PL; (ii) 100 U/mL IL-1α; (iii) 5% PL + 100 U/mL IL-1α; (iv) without any supplement. Cells were washed with PBS to remove residual factors and incubated in serum-free medium. After 24 h, cell conditioned media were collected and replaced with fresh serum-free medium that was collected at 48 h. New serum-free medium was added and collected at 72 h. The three sets of conditioned media were clarified at 2,000 rpm for 10 min at room temperature and stored at −20°C for further analysis. For COX-2 detection, at the end of 24-h stimulation and of 72-h serum-free incubation, cells were washed with PBS, lysed by incubating the cell layers on ice for 5 min with ice-cold RIPA buffer (50 mM Tris HCl pH 7.5, 150 mM NaCl, 1% w/v sodium deoxycholate, 1% v/v Triton X-100, 0.1% w/v sodium dodecyl sulfate and 0.2% w/v sodium azide) supplemented with protease inhibitor cocktail, harvested with cell scrapers, clarified at 10,000 rpm for 15 min at 4°C and stored at −20°C. To study the PL effect on proliferation-related pathways and on cell cycle activation, sub-confluent HS-isolated hASC were starved in serum-free medium for 1 h before being treated with serum-free medium supplemented with 5% PL for different time intervals. Cell layers were washed with cold PBS on ice, frozen at −80°C for at least 1 h, incubated on ice with ice-cold RIPA buffer supplemented with protease inhibitor cocktail and treated as described above.

The protein content was quantified by Bradford protein assay (Bradford, 1976). Electrophoresis was performed in reducing conditions loading 6 μg of total protein/sample for conditioned media and 30 μg or 100 μg of cell lysates on NuPAGE™ 4–12% Bis-Tris gel and transferring to an Amersham™ Protran™ 0.45 μm NC nitrocellulose blotting membrane. The membrane was treated as described in Ulivi et al. (2006). For each considered marker, western blot was performed on 3 independent experiments corresponding to different single-donor primary hASC cultures. The densitometric analysis was performed by quantifying the band densities using ImageJ software (https://imagej.nih.gov/ij/download.html). The reported results are the average of 3 independent experiments ± SD values.

### PGE_2_ Quantification

For the quantification of PGE_2_ in the hASC-conditioned media, Prostaglandin E_2_ ELISA kit was used following manufacturer's instructions. For each culture condition, the reported PGE_2_ concentration is the average value of 3 independent experiments assayed in duplicate on different single-donor primary cultures ± SD.

### *In vitro* Scratch Assay

HS-isolated hASC at P2 were seeded at 1.0 × 10^5^ cells/dish in 60 mm dishes, cultured in complete medium to confluence and treated for 24 h with serum-free medium supplemented with: (i) 5% PL; (ii) 100 U/mL IL-1α; (iii) 5% PL + 100 U/mL IL-1α; (iv) without any supplement. Cells were washed with PBS to remove residual factors and incubated in serum-free medium. After 24 h, cell conditioned media were collected and used for the *in vitro* scratch assay.

NCTC 2544 keratinocytes were plated at 3.0 × 10^4^ cells/well in 48-well plates and maintained in their culture medium until confluence. At confluence, cells were washed twice with PBS and incubated in serum-free αMEM for 24 h. Cell monolayers were scratched using 2–100 μL pipette tips, washed once with PBS and covered with 200 μL/well of media (75% conditioned media, 25% fresh serum-free αMEM). Wells covered with serum-free αMEM were used as internal negative control. After 0- and 24-h incubation, cells were washed with PBS, fixed with 4% (w/v) paraformaldehyde in PBS for 30 min at room temperature and stained with 1% methylene blue solution at pH 8.8 for 30 min at room temperature. Three independent experiments were performed in triplicate using conditioned media from different single-donor primary hASC cultures.

The analysis of scratch closure was performed as reported by Spanò et al. (2018) with some modifications. For each stained cell monolayer, 3 images were captured along the scratch with a 10x objective of a microscope Axiovert 200M (Carl Zeiss). Images were then converted into binary images using ImageJ and were analyzed by TScratch software (https://github.com/cselab/TScratch). For each image, the “open image area” was calculated by TScratch and transformed in percentage value (open area percentage, OAP), corresponding to the percentage of scratched area. For each tested condition, the average OAP (±SD) was calculated at 0 and 24 h and was used to obtain the “closed scratch area,” i.e., the percentage of scratch closed following the treatment with conditioned medium, using the following formula: closed scratch area $=\frac{OAP_{0h}-OAP_{24h}}{OAP_{0h}}\times 100$.

### Histology

After 14 day culture, *in toto* hAT samples were fixed with a formol-calcium solution overnight at 4°C, dehydrated with increasing ethanol concentrations, cleared in xylol and embedded in paraffin. As control, some *in toto* hAT samples were fixed and paraffin-embedded immediately after their isolation from the resection material. All samples were cut into 5 μm sections, which were placed on poly-L-lysine-coated slides and dried overnight at 37°C. For Hematoxylin and Eosin (H&E) staining, deparaffinised sections were stained with Harris hematoxylin for 3 min, counterstained with 0.25% (w/v) eosin Y for 30 sec, dehydrated in upgraded ethanol to xylol and mounted in Eukitt®. For immunohistochemical investigations, deparaffinised sections were incubated with 0.2% Triton in PBS for 10 min, incubated with 0.3% H_2_O_2_ in distilled water for 30 min to block endogenous peroxidase activity and covered with 10% normal goat serum in PBS for 1 h to reduce non-specific staining. Sections were then incubated with primary antibody raised against PCNA (1:200 dilution) diluted in 5% normal goat serum in PBS overnight at 4°C in a humid chamber. As negative control, one section per slide was incubated only with 5% normal goat serum in PBS. For detection of primary antibody, sections were incubated first with biotinylated IgGs (1:200 dilution), diluted in 5% normal goat serum in PBS, and then with streptavidin-peroxidase (1:500 dilution), diluted in 5% normal goat serum in PBS, each for 30 min at room temperature. The peroxidase was visualized using DAB following manufacturer's instructions. Images were acquired by microscope Axiovert 200M (Carl Zeiss). A complete histological analysis was performed on each of 3 different single-donor hAT samples.

### Statistical Analysis

All data are presented as means and standard deviations based on independent experiments performed on at least three different primary hASC cultures, each of them derived from a single donor. The statistical analysis was performed using the unpaired *t*-Test for cell counting and viability assays, real time quantitative PCR analysis and ORO quantification or using the ordinary one-way ANOVA for crystal violet staining assay, CFE assay, IL-6, IL-8, COX-2, STAT3, and Cyclin D1 densitometric analyses, PGE_2_ quantification, and *in vitro* scratch assay. If ANOVA detected statistically significant differences within the data set, Tukey's multiple comparison test was used to calculate the significant differences for IL-6, IL-8, and COX-2 densitometric analyses, PGE_2_ quantification, crystal violet staining assay, CFE assay and for *in vitro* scratch assay. Dunnett's multiple comparison test was used for STAT3 and Cyclin D1 densitometric analyses. All tests were run setting a confidence interval of 95%.

## Results

### HS Sustains hASC Proliferation More Efficiently Than FBS and Induces Spontaneous Adipogenic Differentiation in the Cultured Cells

**Figure 1:**
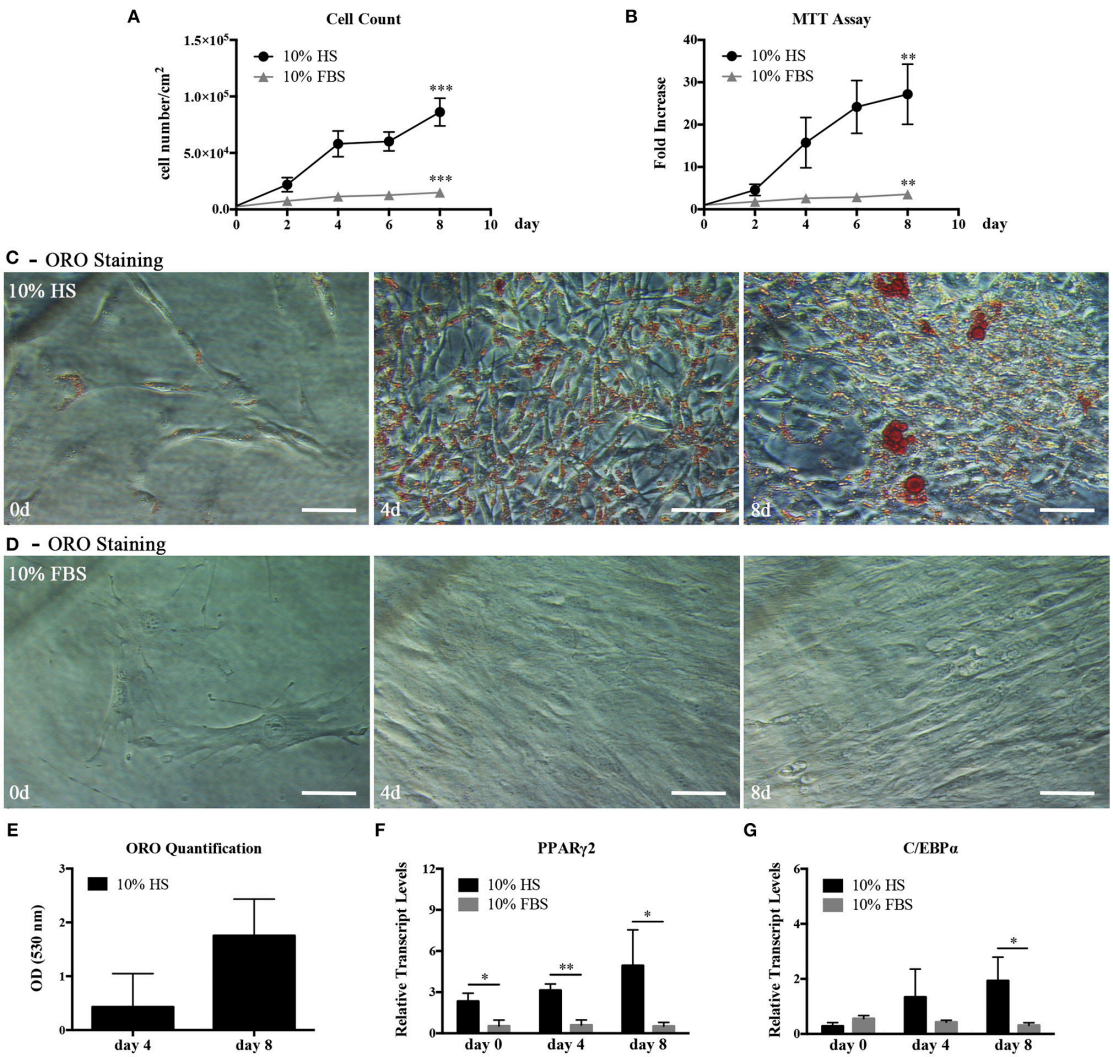
hASC isolated and expanded in 10% HS or 10% FBS at passage 2. **(A)** Cell proliferation monitored by cell counting. Average values ± SD of 3 independent experiments, each one performed in duplicate, are reported. The ^***^ symbol corresponds to *p* = 0.0006; **(B)** Cell viability evaluated by MTT assay and expressed as n-fold increase. Average values ± SD of 3 independent experiments, each one performed in triplicate, are reported. The ^**^ symbol corresponds to *p* = 0.005; **(C,D)** Representative ORO staining of hASC cultured with 10% HS **(C)** or 10% FBS (D) for 0, 4, and 8 days (scale bars = 50 μm); **(E)** Quantification by spectrophotometric analysis of ORO dye incorporated in HS-expanded hASC, normalized to cell number, at different culture days. Average values ± SD of 3 independent experiments, each one performed in triplicate, are reported; **(F,G)** Real Time quantitative PCR analysis of PPARγ2 **(F)** and C/EBPα **(G)** transcript levels in HS- or FBS-expanded hASC at different culture days. Results are expressed as means ± SD values of 3 independent experiments performed each in triplicate. The ^*^ and ^**^ symbols refer to *p* ≤ 0.04 and *p* = 0.002, respectively.

We, first, examined the proliferation and differentiation capabilities of hASC expanded in 10% HS in comparison with the same cells expanded in the presence of 10% FBS. The HS-expanded hASC had a higher proliferation rate than FBS-expanded cells reaching a significant increase in the cell density after 8 day culture (5.75 ± 1.35 fold; *p* = 0.0006. Figure 1A). In a parallel set of experiments, we also evaluated cell viability by MTT assay at different culture times and obtained comparable results (Figure 1B). It is to note that, we did not find a significant difference in the number of colony-forming unit-fibroblasts (CFU-f) between HS- and FBS-supplemented medium by CFE assay suggesting that the increased number of cells was due to an increase of the proliferation rate and not to an increased number of isolated proliferating cells (see FBS and HS controls in **Figures 4A,B**). Variations in cell morphology were observed in the two culture conditions. In particular, HS-expanded hASC were elongated and smaller than FBS-expanded cells, which showed a spreading and a flatter shape (Figures 1C,D).

In hASC cultured in the presence of HS but not in the presence of FBS, we observed within the cells spherical droplets increasing in number and dimension with culture time. These droplets were red-stained by the lipid-specific ORO staining (Figures 1C,D). Quantification of incorporated ORO dye, normalized to cell number, confirmed the progressive increase of intracellular lipid deposits (Figure 1E). In agreement with the above observations, the analysis of the total RNA extracted from hASC cultured with either HS or FBS for 0, 4, and 8 days revealed that the transcript levels of PPARγ2 and C/EBPα, two master regulators of adipogenesis, gradually increased in HS-expanded hASC. On the contrary, the two transcripts remained at a basal level in FBS-expanded hASC (Figures 1F,G). After 8-day culture, PPARγ2 and C/EBPα transcript levels in HS-expanded cells reached a level significantly higher than in FBS-expanded cells (*p* = 0.04 and *p* = 0.03, respectively).

### Induction of Adipogenic and Osteogenic Differentiation in hASC Expanded in the Presence of HS

**Figure 2:**
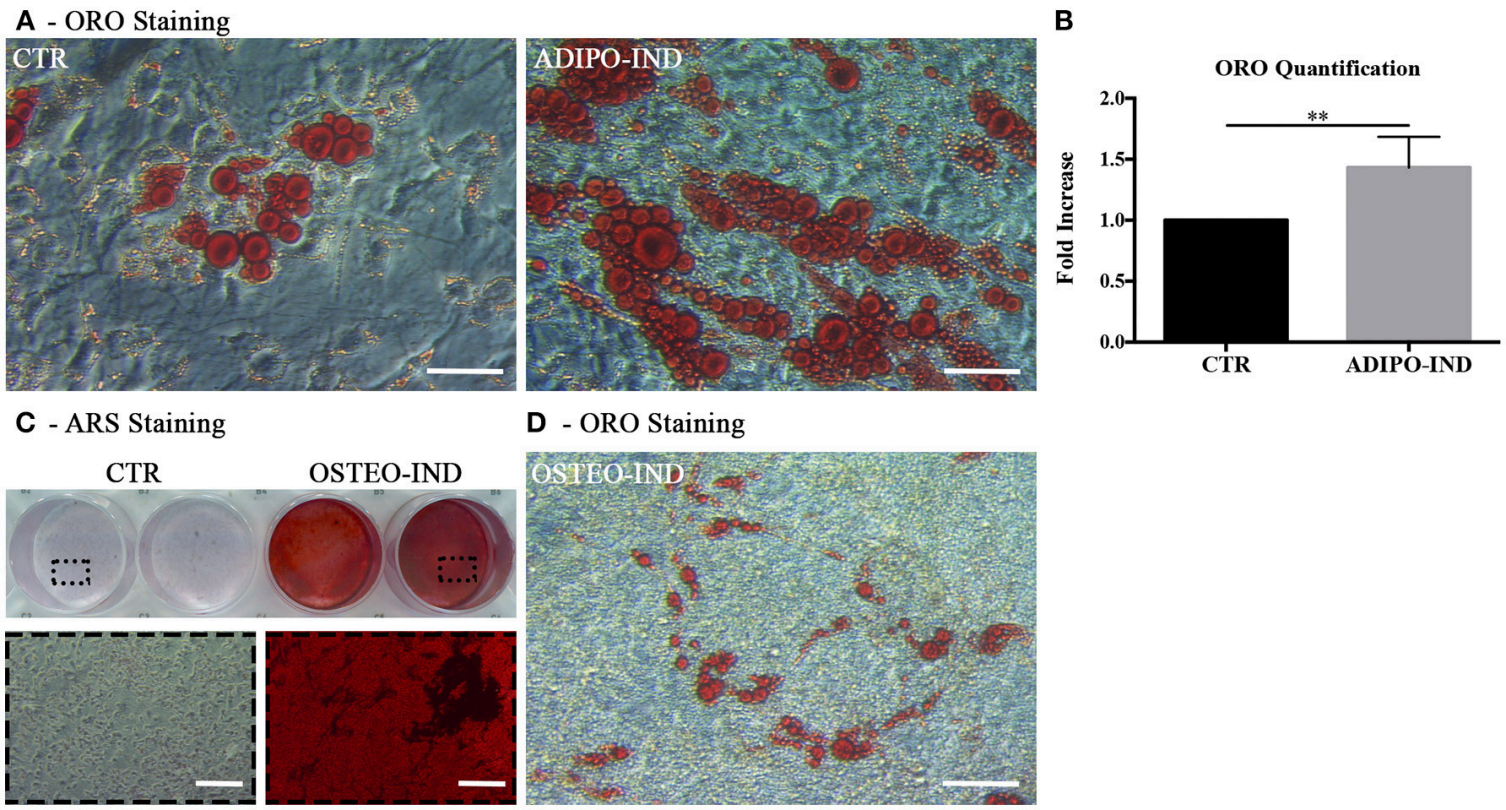
Induction of adipogenic and osteogenic differentiation of HS-expanded hASC. **(A)** Representative ORO staining of adipogenesis-induced and control cells (scale bars = 50 μm); **(B)** Quantification by spectrophotometric analysis of ORO dye incorporated in adipogenesis-induced and control (CTR) cells. Results are expressed as ratio referred to CTR value. Average values ± SD of 6 independent experiments, each one performed in triplicate, using cells either at passage 2 (*n* = 3) or 5 (*n* = 3) are reported. The ^**^ symbol corresponds to *p* = 0.002; **(C)** Representative ARS staining of osteogenesis-induced and control cells. Lower images are magnifications of the highlighted areas (scale bars = 200 μm); **(D)** Representative ORO staining of osteogenesis-induced cells (scale bars = 50 μm).

In light of the above results, unless differently indicated, hASC were cultured in the presence of HS in all following experiments. In the ORO-stained cell layers of high-density hASC cultured for 8 days in the presence of HS, we noticed the presence of several areas with ORO-negative cells. To investigate the capability of these ORO-negative cells to differentiate toward adipocytes, we treated sub-confluent hASC with a standard adipogenic differentiation medium. After 2-week induction, the amount of incorporated ORO in the induced cells significantly increased with regard to not-induced control cells (*p* = 0.002; Figures 2A,B). However, several ORO-negative cells were still present in the induced cell layers.

When the HS-expanded hASC from the same cultures were osteogenically induced for 2 weeks, we observed a great amount of calcium-rich deposits in the extracellular matrix of induced cells, as revealed by ARS staining (Figure 2C). Interestingly, we observed the persistence of cells containing ORO-stained lipid droplets in the cell layers of these osteogenesis-induced hASC (Figure 2D). It is to note that, when a chondrogenic differentiation was induced, the pellet cultures of HS-expanded hASC presented a poor metachromatic toluidine blue staining and no collagen type II was detected in the extracellular matrix by immunohistochemistry (Supplementary Figure 1 and Supplementary Material).

### PL Enhances Proliferation of HS-Expanded hASC

**Figure 3:**
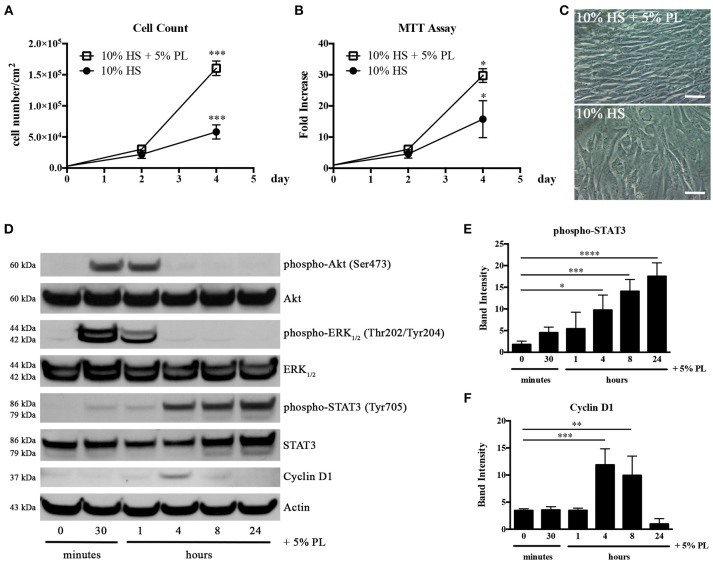
Effect of PL on proliferation of HS-expanded hASC at passage 2. **(A)** Cell proliferation monitored by cell counting. Average values ± SD of 3 independent experiments, each one performed in duplicate, are reported. The ^***^ symbol corresponds to *p* = 0.0004; **(B)** Cell viability evaluated by MTT assay and expressed as n-fold increase. Average values ± SD of 3 independent experiments, each one performed in triplicate, are reported. The ^*^ symbol corresponds to *p* = 0.02; **(C)** Morphology of PL-stimulated and un-stimulated cells after 4-day treatment (scale bars = 50 μm); **(D)** Representative western blots of total protein content in lysates of hASC stimulated with PL, in the absence of HS, for different times. Primary antibodies were raised against phospho-Akt, Akt, phospho-ERK_1/2_, ERK_1/2_, phospho-STAT3, STAT3, Cyclin D1, and against Actin used as internal control; **(E,F)** Densitometric analyses (means ± SD) performed on 3 different phospho-STAT3- **(E)** and Cyclin D1-probed **(F)** western blots deriving from three independent experiments. The ^*^, ^**^, ^***^, and ^****^ symbols refer to *p* = 0.02, *p* = 0.007, *p* ≤ 0.0009, and *p* < 0.0001, respectively.

Platelets release a cocktail of bioactive molecules by degranulation as soon as an injury occurs. In order to mimic the wound microenvironment, we evaluated the effect of 5% PL on proliferation and viability of hASC isolated and cultured in HS. The presence of PL in the complete medium induced a significant increase of both cell proliferation and viability after 4-day treatment (*p* = 0.0004 and *p* = 0.02, respectively; Figures 3A,B). In particular, the average cell density was 2.8 ± 0.6-fold higher with respect to the PL un-supplemented control cultures. The PL treatment determined also an immediate change in the morphology of the cells that assumed a spindle-like shape and became smaller than control cells maintained in the absence of PL (Figure 3C).

In parallel experiments, HS-isolated hASC were serum starved for 1 h before treatment with serum-free medium supplemented with 5% PL. A direct effect of PL on the activation of the cell proliferation machinery was confirmed by the strong and transient phosphorylation of Akt and ERK_1/2_ starting from 30 min after PL stimulation and already decreasing after 1 h (Figure 3D). We observed an analogous activation of these pathways also in cell lysates of hASC treated with PL in the presence of complete medium containing HS (data not shown). PL induced also a significant transient increase in the Cyclin D1 expression. The highest amount of Cyclin D1 was observed after 4 h of PL treatment (*p* = 0.0009 for 0 vs. 4 h) and was already decreased after 8 h treatment (Figures 3D,F). An analogous trend in the Cyclin D1 synthesis was observed also when hASC were treated with PL in the presence of HS (data not shown).

Interestingly the PL treatment also induced the activation of the STAT3 pathway. This activation was gradual starting from 30 min and reached its maximum level at 24 h (*p* < 0.0001 for 0 vs. 24 h; Figures 3D,E).

**Figure 4:**
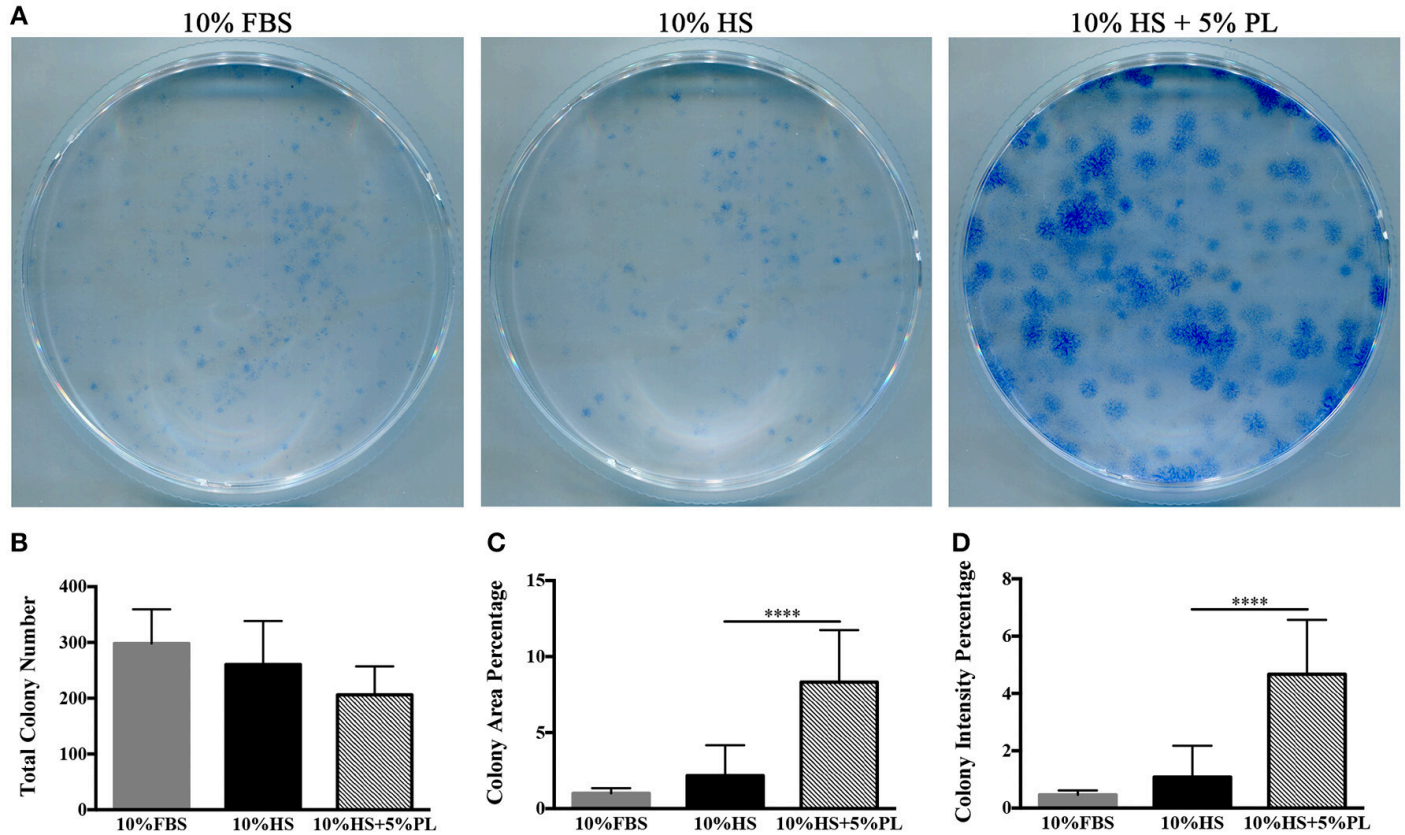
CFE assay performed on hASC immediately after their isolation (passage 0) by using media supplemented with 10% FBS, 10% HS, or 10% HS + 5% PL. **(A)** Scans of methylene blue-stained colonies deriving from a representative experiment; **(B)** Number of colonies per condition counted by naked eye. Average values ± SD of 5 independent experiments, each one performed in duplicate; **(C,D)** “Colony area percentage” and “colony intensity percentage” calculated by ImageJ-plugin “ColonyArea.” Average values ± SD of 5 independent experiments, each one performed in duplicate. The ^****^ symbol corresponds to *p* < 0.0001.

To confirm that the increase in the number of cells was due to an increased proliferation rate and not to an increased number of isolated proliferating cells, we performed some cloning experiments. In the presence of PL, the number of CFU-f did not significantly vary compared to un-supplemented culture condition (Figures 4A,B). Instead, both “colony area percentage” and “colony intensity percentage,” calculated by ImageJ-plugin “ColonyArea,” were significantly higher with PL-supplemented complete medium than with un-supplemented medium confirming the increased cell proliferation rate in the presence of PL (*p* < 0.0001; Figures 4C,D).

### PL-Stimulated hASC Reversibly Lessen the Adipogenic Phenotype

**Figure 5:**
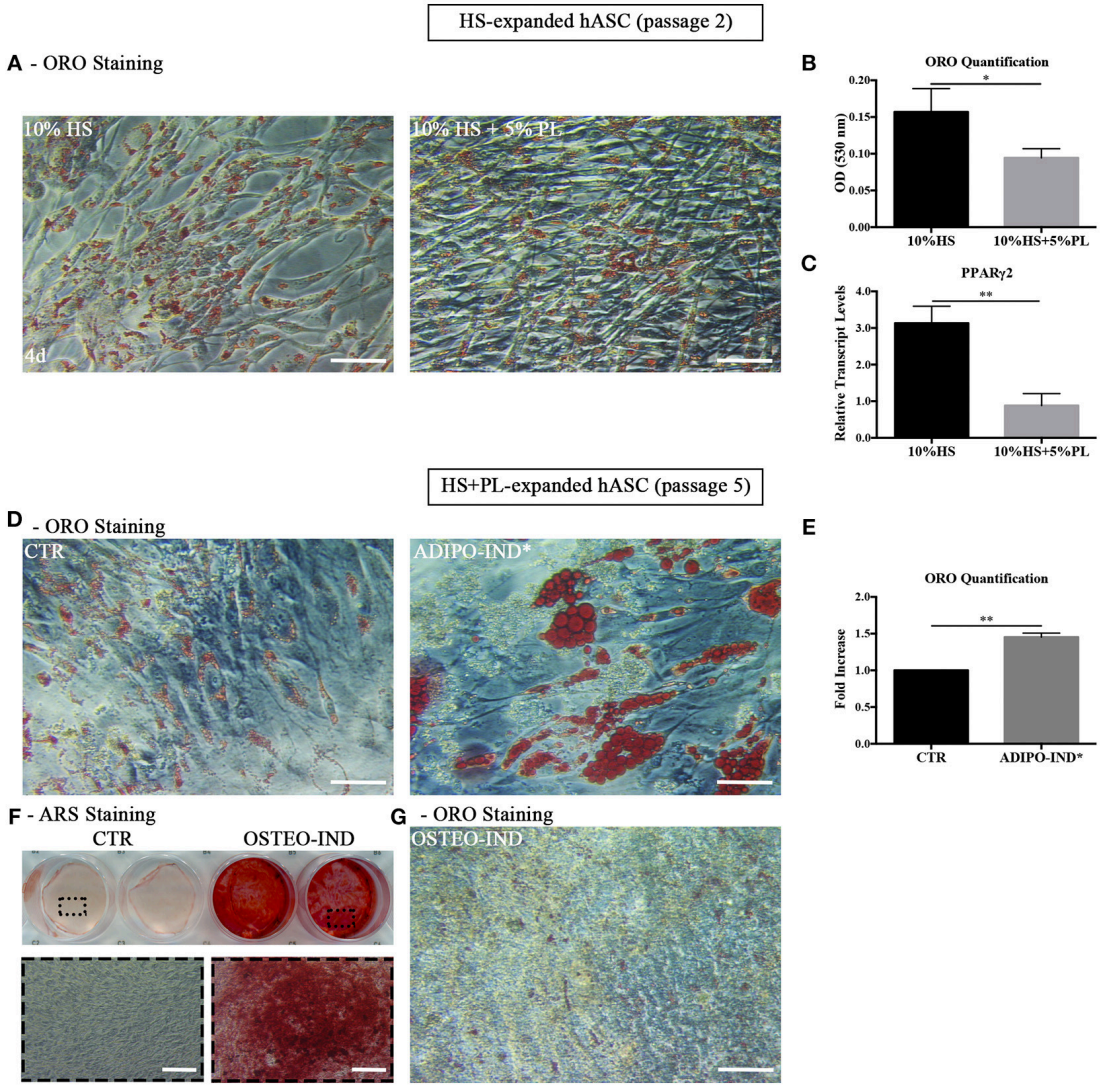
**(A,B)** Differentiation potential of HS-expanded hASC after PL stimulation. **(A)** Representative ORO staining of PL-stimulated and un-stimulated cells at passage 2 after 4-day treatment (scale bars = 50 μm); **(B)** Quantification by spectrophotometric analysis of ORO dye incorporated in PL-stimulated and un-stimulated hASC, normalized to the cell number ratio between HS and HS+PL. Average values ± SD of 4 independent experiments, each one performed in triplicate and corresponding to 3 different hASC cultures, are reported. The ^*^ symbol refers to *p* = 0.01; **(C)** Real Time quantitative PCR analysis of PPARγ2 transcript level in cells treated or not treated with PL for 4 days. Results are expressed as means ± SD values of 3 independent experiments assayed in triplicate. The ^**^ symbol refers to *p* = 0.002; **(D–F)** HS-expanded hASC cultured in the presence of 5% PL for additional 3 passages were induced toward adipogenesis and osteogenesis. **(D)** Control and adipogenesis induced cells ORO staining (scale bars = 50 μm); **(E)** Quantification by spectrophotometric analysis of ORO dye incorporated in adipogenesis-induced and control (CTR) cells. Results are expressed as ratio referred to CTR value. Average values ± SD of 2 independent experiments, each one performed in triplicate, are reported. The ^**^ symbol corresponds to *p* = 0.008; **(F)** Representative ARS staining of osteogenesis-induced and control cells. Lower images are magnifications of highlighted areas (scale bars = 200 μm); **(G)** Representative ORO staining of osteogenesis-induced cells (scale bars = 50 μm).

Given the spontaneous adipogenic differentiation of HS-expanded hASC and their response to the adipogenic and osteogenic differentiation induction, we studied the differentiation potential of the same cells also after PL stimulation. The hASC expanded with HS for two culture passages and stimulated with 5% PL for 4 days retained the capability to differentiate spontaneously toward adipogenesis, as revealed by ORO staining (Figure 5A), but the amount of incorporated ORO, normalized to the cell number, significantly decreased in PL-treated cells (*p* = 0.01; Figure 5B). At the same time, we also observed a significant decrease of PPARγ2 transcript level in PL-treated cells compared to un-treated cells (*p* = 0.002; Figure 5C).

However, when we expanded HS-isolated hASC with complete medium supplemented with 5% PL for further 3 passages and induced them to differentiate toward adipogenesis by using the standard differentiation medium supplemented with indomethacin and IBMX, we observed in the induced cells the presence of intracellular ORO-stained lipid droplets larger than those observed in the control un-induced culture (Figure 5D). The amount of incorporated ORO in the induced cells significantly increased compared to the control cells (*p* = 0.008; Figure 5E). The same cells, when osteogenically induced, produced a mineralized ARS-stained extracellular matrix in response to the induction (Figure 5F). As observed before, ORO-positive lipid droplets were still present within osteogenesis-induced hASC (Figure 5G) even though lipid droplets were fewer and smaller compared to the control cells (Figure 5A). The data suggest that the effect of PL is to lessen the adipogenic phenotype in the cell population without affecting the capacity of the cells to revert to adipocytes when they are transferred to a permissive environment.

### PL Exerts a Mitogenic Effect in Both the Presence and the Absence of an Inflammatory Milieu

**Figure 6:**
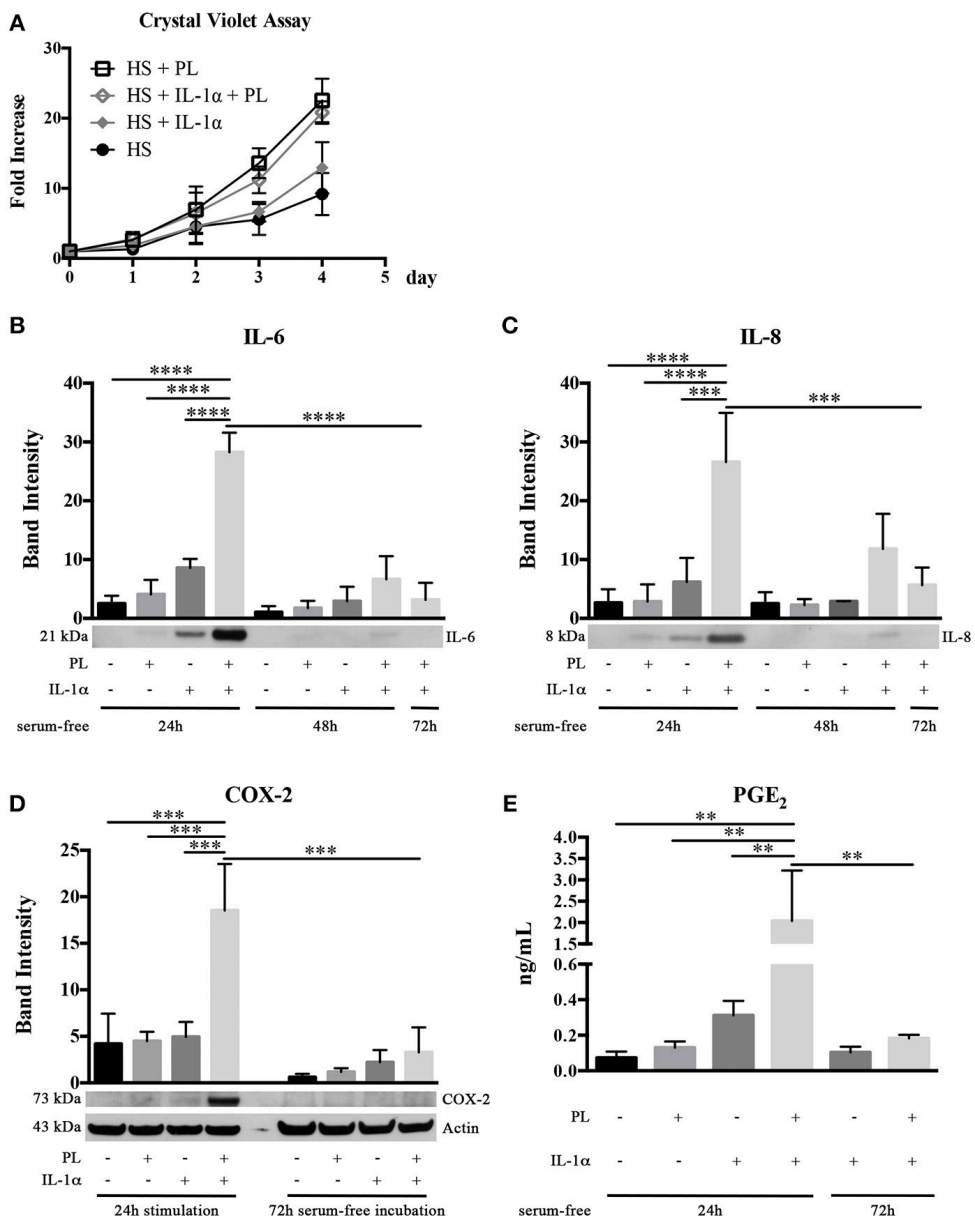
Effect of PL on proliferation and inflammatory response of HS-expanded hASC at passage 2 within an inflammatory milieu. **(A,B)** Cell proliferation monitored by crystal violet staining assay in normal and inflammatory (i.e., in the presence of IL-1α) conditions for different times **(A)**; **(B,C)** Secretion of IL-6 **(B)** and IL-8 **(C)** in hASC-conditioned media collected 24, 48, and 72 h after 24-h PL stimulation, in presence or absence of IL-1α. Densitometric analyses (means ± SD) of the western blots from 3 independent experiments and representative western blots (in the lower panels) are reported. The ^***^ and ^****^ symbols correspond to *p* = 0.0002 and *p* < 0.0001, respectively; **(D)** COX-2 production in hASC-cell lysates harvested after 24-h PL stimulation, in presence or absence of IL-1α, and after further 72-h serum-free incubation. Densitometric analysis (means ± SD) of the western blots from 3 independent experiments and representative western blots (in the lower panels) are reported. Actin was used as internal control. The ^***^ symbol corresponds to *p* ≤ 0.0009; **(E)** Quantification by ELISA of PGE_2_ in hASC-conditioned media collected 24 and 72 h after 24-h PL stimulation, in presence or absence of IL-1α. Average values of 3 independent experiments performed in duplicate ± SD values are reported. The ^**^ symbol refers to *p* ≤ 0.003.

Having in mind that, at the wound site, the recruitment of stem/progenitor cells out from their tissue niche takes place in an inflammatory milieu, we investigated the PL effect on hASC growth not only in the absence, but also in the presence of the inflammatory cytokine IL-1α. We confirmed the enhanced proliferation of HS-expanded hASC due to the PL supplement, but we did not observe any significant difference between cells cultured in medium supplemented with either HS+PL or HS+PL+IL-1α (Figure 6A). Similarly no differences were also observed in control cells supplemented with either HS or HS+IL-1α (Figure 6A).

### In the Presence of an Inflammatory Milieu, PL Induces a Transient Pro-Inflammatory Response in hASC

Considering that, at the wound site, the platelet content is also playing a role in determining and modulating the inflammatory state, we investigated in an inflammatory milieu (mimicked by the presence of IL-1α) the PL effect on the secretome of hASC. Sub-confluent hASC were treated for 24 h with serum-free medium containing 100 U/mL IL-1α and either supplemented or not supplemented with 5% PL. Control cultures with and without PL in the absence of IL-1α were performed in parallel. At the end of the 24-h stimulation, the media were replaced every 24 h with fresh serum-free medium and conditioned media collected at 24, 48, and 72 h. By western blot analysis, we observed a significantly increased secretion of pro-inflammatory cytokines IL-6 and IL-8 in the 24-h medium conditioned by cells stimulated with PL+IL-1α compared to IL-1α (*p* < 0.0001 and *p* = 0.0002, respectively). IL-6 and IL-8 secretion in PL+IL-1α gradually decreased to basal level after 72 h (Figures 6B,C). Moreover, in a parallel set of experiments, cell lysates were harvested at the end of the 24-h stimulation and after additional 72 h of serum-free incubation. By western blot analysis, we observed a strongly increased production of COX-2 after 24 h of PL and IL-1α treatment compared to only IL-1α or PL (*p* = 0.0009 and *p* = 0.0007, respectively) and the complete disappearance of the signal after 72 h (Figure 6D). In agreement to the COX-2 induction, we found a significantly increased concentration of PGE_2_ in the 24-h medium conditioned by PL+IL-1α-stimulated cells compared to only IL-1α or PL (*p* = 0.002 and *p* = 0.003, respectively), indicating a synergistic activity of IL-1α and PL (Figure 6E). After 72-h serum-free incubation, PGE_2_ levels decreased and returned to the basal level.

### Media Conditioned by the PL-Stimulated hASC Exert a Chemotactic Activity on Human Keratinocytes and Favor the Healing of an *in vitro* Scratch Wound

**Figure 7:**
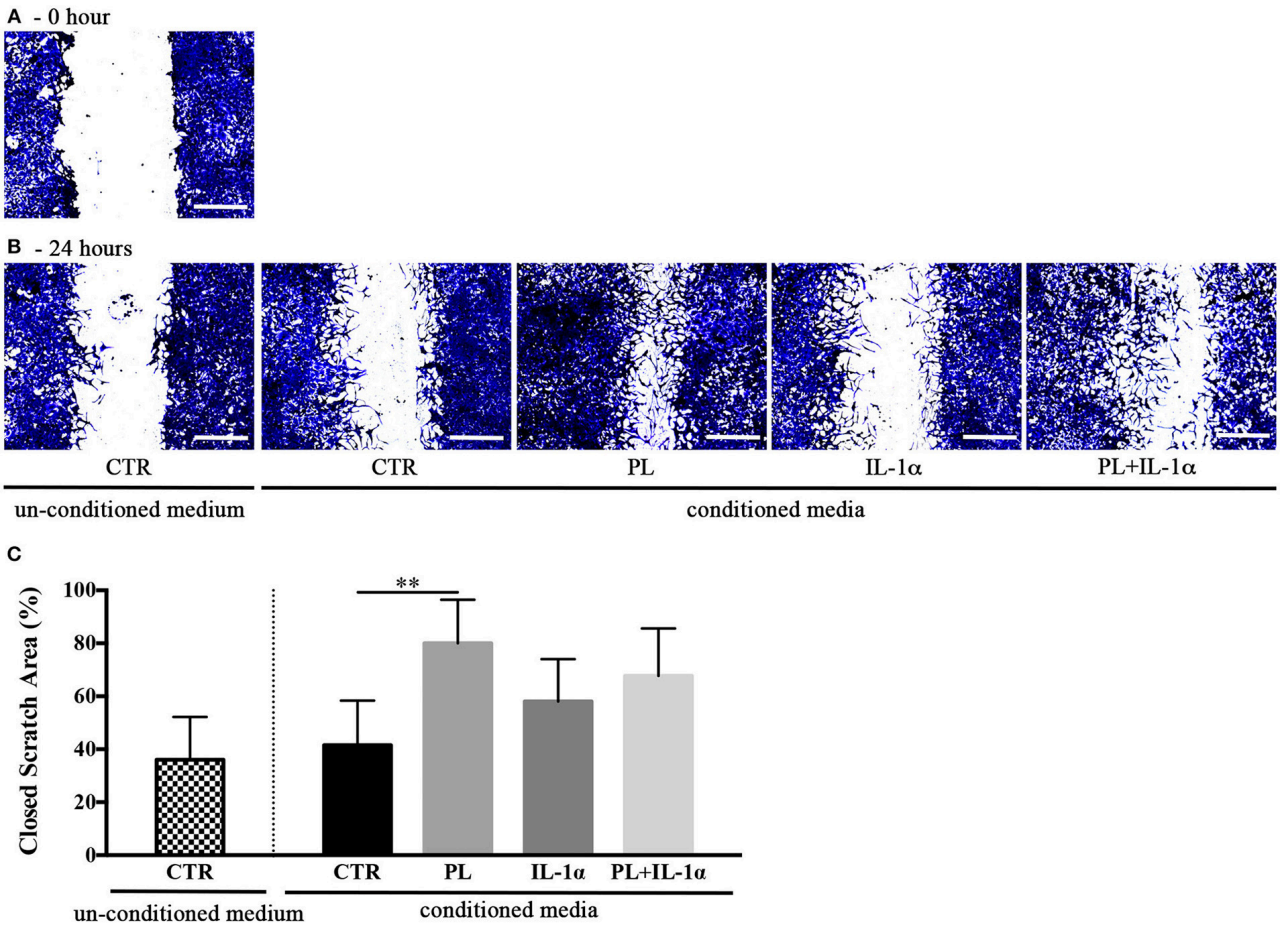
Effect of hASC-conditioned media, collected 24 h after 24-h PL stimulation in presence or absence of IL-1α, on keratinocyte migration evaluated by an *in vitro* scratch assay. **(A,B)** Representative images of scratched confluent cell monolayers incubated with 75%-diluted hASC-conditioned media for 0 **(A)** and 24 h **(B)** (scale bars = 300 μm). CTR refers to serum-free medium. Supplemented and un-supplemented conditioned media were collected 24 h after a previous 24-h serum deprivation; **(C)** “Closed scratch area” calculated by TScratch software at the end of incubation in all tested conditions. Average values ± SD of 3 independent experiments, each one performed in triplicate. The ^**^ symbol corresponds to *p* = 0.004.

Given the hypothesis that PL-stimulated subcutaneous adipose tissues could participate to skin repair, we investigated by an *in vitro* scratch assay the effect on human keratinocyte migration of the serum free media conditioned for 24 h by hASC previously stimulated for 24 h by PL in either the presence or the absence of IL-1α. The scratches treated with media conditioned by PL-stimulated hASC displayed a significant increase of the closed scratch area with respect to control conditioned medium (*p* = 0.004; Figure 7). Media conditioned by PL+IL-1α-stimulated hASC induced an acceleration, but not significant, of the wound closure compared to the controls.

### PL Induces Cell Proliferation Also in the *in toto* Human Adipose Tissue (hAT) Cultured *ex vivo*

**Figure 8:**
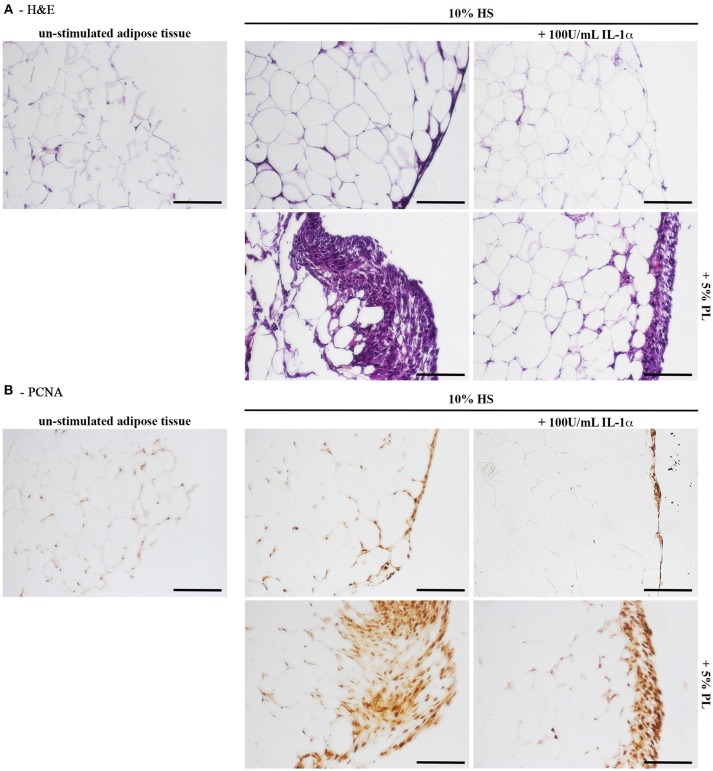
Histology of *in toto* hAT samples cultured *ex vivo* using medium containing HS without any other supplement or supplemented with 5% PL, 100 U/mL IL-1α, or 5% PL + 100 U/mL IL-1α, for 14 days. “Un-stimulated adipose tissue” refers to hAT samples paraffin-embedded immediately after their isolation from the resection material and not cultured. **(A)** Hematoxylin and Eosin staining of paraffin-embedded hAT cross sections (scale bars = 150 μm); **(B)** Immunohistochemistry of paraffin-embedded hAT cross sections with a specific primary antibody raised against the nuclear proliferation marker PCNA (scale bars = 150 μm).

**Figure 9:**
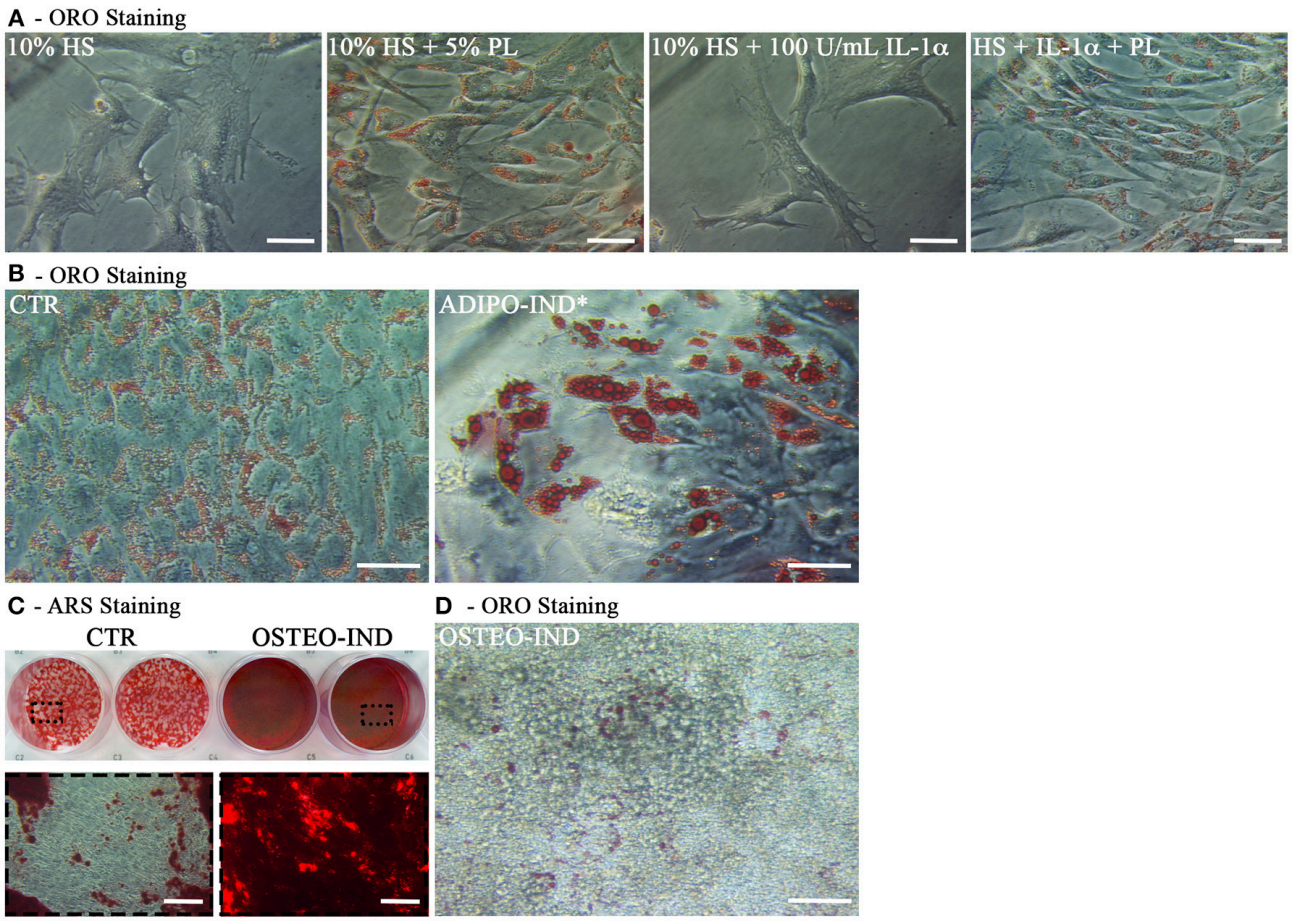
Characterization of cells outgrown from *in toto* hAT samples cultured *ex vivo* using complete medium supplemented with 5% PL, 100 U/mL IL-1α, or 5% PL + 100 U/mL IL-1α, or without any supplement, for 14 days. **(A)** Representative ORO staining of outgrown cells at passage 0 in all different culture conditions (scale bars = 50 μm); **(B)** Representative ORO staining of cells outgrown from the HS+PL-stimulated hAT, cultured for 3 passages in HS-supplemented medium, in the absence of PL, and induced or not induced toward adipogenesis (scale bars = 50 μm); **(C)** Representative ARS staining of cells outgrown from the HS+PL-stimulated hAT, cultured for 3 passages in HS-supplemented medium, in absence of PL, and induced or not induced toward osteogenesis. Lower images are magnifications of highlighted areas (scale bars = 200 μm); **(D)** Representative ORO staining of osteogenesis-induced cells (scale bars = 50 μm).

Next to the *in vitro* experiments with cultured hASC, we performed *ex vivo* cultures of *in toto* small biopsies of human adipose tissue from 5 donors. The biopsies were maintained for 2 weeks in the complete medium supplemented with either 5% PL, or 100 U/mL IL-1α, or 5% PL + 100 U/mL IL-1α, or without any supplement. We did not observe any detectable change in morphology and phenotypic appearance of the hAT in all cultures. Under all conditions, we observed spindle-like cells to come out from the samples, to adhere to the plastic and to proliferate. This cell outgrowth was particularly evident in the PL stimulated cultures (**Figure 9A**). After 2 weeks, hAT samples were collected, paraffin-embedded and examined by histology. The H&E staining revealed the presence of small and elongated cells in the outer layer of the samples. Once again the presence of these elongated small cells was particularly evident in the PL-supplemented cultures (Figure 8A). Moreover, the immunohistochemical analysis with a specific antibody directed against the proliferation marker PCNA indicated that the small outlying cells were actively proliferating (Figure 8B).

### Cells Out-Grown From PL-Cultured hAT Maintain an Adipogenic and Osteogenic Differentiation Potential

At the end of the hAT culture, ORO staining of outgrown cells revealed that cells activated by PL, both in the presence and the absence of IL-1α, displayed intracellular ORO-positive lipid droplets (Figure 9A). This finding suggested that these cells could be adipogenic progenitors. To corroborate this hypothesis, we cultured cells outgrown upon HS+PL stimulation for 3 passages in complete medium, in the absence of PL, and induced them toward adipogenesis by using the standard differentiation medium supplemented with indomethacin and IBMX. After 2-week induction, the ORO staining revealed the presence in the cells of lipid droplets much bigger than in the control cells (Figure 9B). The same cells were also induced toward osteogenic differentiation obtaining a strongly mineralized ARS-stained extracellular matrix (Figure 9C). Also un-induced control cells showed some ARS staining, suggesting that adherent cells undergo by default osteogenic differentiation. As reported before, we observed ORO-positive lipid droplets within cells of osteogenesis-induced cultures, albeit fewer and smaller than in control cultures (Figure 9D).

## Discussion

A wound-healing process is a sequence of finely orchestrated events leading to the repair of the damaged tissue. The present interest of our research group is the understanding of the molecular basis of the wound-healing process in order to identify endogenous pathways naturally activated following an injury and to envisage therapeutic approaches to restore the impaired healing. Following our previous work on the platelet-lysate effects on human keratinocytes (El Backly et al., 2011), we studied the response of human subcutaneous adipose tissue to platelet-derived factors under conditions mimicking, as much as possible, the wound microenvironment in order to evaluate the adipose-tissue contribution in supporting skin lesion repair. For this purpose, we defined a clinically relevant model using Human Serum, Platelet Lysate and Interleukin-1α as effectors, and primary hASC and *in toto* hAT cultured *ex vivo* as targets. It is to note that, because of the adopted manufacturing procedures, the HS was free of platelet-derived factors and the PL did not contain plasmatic molecules (Muraglia et al., 2017). Therefore, we were able to study the separated and combined effects of plasma molecules and platelet content on adipose tissue.

Since serum is the physiologic fluid formed at the wound site at the beginning of the healing, during the coagulation process, we first evaluated the effect of a human platelet-free serum on *in vitro* proliferation and differentiation of hASC in comparison with FBS, obtained from whole bovine blood and considered the gold standard as medium supplement for hASC cultures. We found that HS sustained *in vitro* hASC expansion with a growth rate significantly higher than FBS, in agreement with other literature data (Kocaoemer et al., 2007; Hui et al., 2012; Witzeneder et al., 2013). As reported also by Kocaoemer et al. (2007), no significant differences were observed in the number of CFU-f isolated in the presence of HS or FBS, thus confirming that the higher number of cells obtained with HS as medium supplement was due to a higher proliferation rate of the cells and not to a higher number of recruited progenitors. Interestingly, we observed that, at variance with FBS, HS favored a spontaneous adipogenic differentiation of the cultured hASC, including the expression induction of two master regulators of adipogenesis, PPARγ2 and C/EBPα, and the accumulation of lipid droplets inside the cells. This observation was not made in previous studies in which FBS was replaced by a human serum (see as example: Kocaoemer et al., 2007; Lindroos et al., 2010), possibly because their sera were prepared from total blood and not from plasma devoid of platelets, as in our HS-production protocol. In agreement, a decrease in the expression of stemness-related genes together with an increase of the cell proliferation induced by human serum was reported by Hui et al. (2012).

However, a significant increase of adipogenic differentiation of HS-expanded cells was induced in permissive conditions whereas, by inducing an osteogenic differentiation in the same cell cultures, a strong deposition of mineralized matrix was observed. However, in the osteogenesis-induced culture, lipid droplets, stainable by ORO dye, were still present within several cells. These findings suggest that HS-expanded hASC are a heterogeneous cell population constituted by progenitor cells at different stages of commitment. In this scenario, we speculate that, in the presence of human plasmatic molecules, more committed cells would continue their adipogenic differentiation process, whereas more immature and less committed cells would respond to exogenous stimuli differentiating toward either the osteogenic or the adipogenic lineage, depending on the stimulus nature. Hypothetically, HS could exert a “niche” effect on hASC regulating self-renewal and differentiation of stem/progenitor cells included in this cell population. Interestingly, very poor or no differentiation toward chondrogenesis was observed at variance with the reports in literature for hASC expanded in FBS (Zuk, 2002), possibly because HS-expanded cells were more committed to adipogenesis.

The coagulation process determines not only the conversion of plasma to serum, but also the release of bioactive molecules from activated platelets by degranulation. To mimic more closely the wound microenvironment, we studied the behavior of hASC also in the presence of the platelet content. The addition of PL to the culture medium already supplemented with HS significantly enhanced cell growth, with a higher cell density at the confluence state, and determined spindle-like morphology and smaller dimensions of the cells as in the case of bone marrow-stromal cells stimulated with PL (Chevallier et al., 2010). Interestingly, the PL determined also in quiescent hASC the early activation of the proliferation-related Akt and ERK pathways (Chambard et al., 2007; Manning and Cantley, 2007) and the expression of Cyclin D1 independently of the contemporary presence of HS. Taken together, these data suggest that platelet-derived factors could activate and induce to proliferate the quiescent progenitor cells also at the wound site. In addition, STAT3, a protein related to cell growth, differentiation and survival signals (Hirano et al., 2000), was also activated. This is an interesting finding because the STAT pathway was reported to be involved in adipose tissue metabolism (Dodington et al., 2018).

As reported by Ruggiu et al. (2013) for osteoblasts, apparently, IL-1α was not playing a role in cell proliferation. Interestingly, we observed that cells cultured in the presence of PL showed a significant decrease of lipid droplets in parallel with a significant decrease of the PPARγ2 transcript level. Taken together, our data suggest that PL treatment would favor the presence of less committed progenitor cells in hASC cultures. In particular, PL could induce the de-differentiation of cells already adipogenic committed or more specifically promote the proliferation of less committed cells. Moreover, hASC expanded repeatedly in the presence of PL retained differentiation capability toward adipogenesis and osteogenesis when cultured in permissive conditions.

In the human body, the adipose tissue is not simply an inert tissue for energy storage, but it is also an endocrine organ producing soluble mediators, named adipokines, which are involved in the regulation of many physiological and pathological processes (Kershaw and Flier, 2004). By using mouse models, Schmidt and Horsley (2013) demonstrated that mature adipocytes repopulated the skin wounds in parallel with fibroblast migration during the proliferative phase of healing. Moreover, they reported evidence of a direct intercellular communication between adipocytes and fibroblasts that might contribute to fibroblast migration during dermal healing. To evaluate an effective contribution of adipose tissue to skin repair, we first investigated the secretory and inflammatory response of HS-expanded hASC to PL in the presence and in the absence of the inflammatory cytokine IL-1α. Under these conditions, we observed a strong synergistic effect of the inflammatory milieu, represented by IL-1α, and the PL stimulation due to the increase of pro-inflammatory cytokine IL-6 and IL-8 secretion, in line with our previous observations in other cell systems (El Backly et al., 2011; Pereira et al., 2013; Ruggiu et al., 2013). This an interesting finding because IL-1α is considered an “alarmin” released by necrotic cells that contribute to active inflammation inducing the release of pro-inflammatory cytokines and that trigger the migration of inflammatory monocytes releasing growth factors, in turn leading to the tissue repair (Kim et al., 2013). Considering that *in vivo* the release of platelet-derived molecules occurs as a burst and is limited in time, we monitored the level of the secreted pro-inflammatory cytokines up to 72 h after the PL removal and we noticed a gradual decrease of their secretion. The production of COX-2, a key enzyme in the inflammatory process (Chen, 2010), was also transiently enhanced by a 24-h burst of PL in the presence of IL-1α. The COX-2 increase led to a massive production and secretion in the medium of PGE_2_, a prostaglandin inducing the functional switch of macrophages toward the alternatively activated M2 phenotype, involved in the inflammation dampening (Tasso et al., 2013; Vasandan et al., 2016). Taken together, the strong but temporally transient increase of IL-6 and IL-8, the induction of COX-2 and the production of PGE_2_ suggest that, during the body response to an injury *in vivo*, the subcutaneous adipose tissue, after being exposed to the platelet-released molecules in an inflammatory milieu, contribute first in establishing the acute inflammatory state and subsequently in promoting the inflammation resolution. We also investigated the global effect of hASC-conditioned media on keratinocyte migration and observed an accelerated rate of closure of *in vitro* scratch wounds by medium conditioned by PL-stimulated cells.

To bridge the gap between *in vitro* results and *in vivo* events, we cultured *ex vivo* hAT biopsies in the presence of HS under normal or inflammatory condition and evaluated the activity of PL in this more complex system. The culture of *in toto* hAT showed that PL induced the proliferation of small and elongated cells on the surface of hAT samples under both physiological and inflammatory conditions, while the presence of these cells was limited in the absence of PL. Moreover, PL induced the release of committed cells adhering to the plastic and proliferating. These cells, cultured in the absence of PL, showed similarly to hASC, the ability to differentiate toward adipogenesis and osteogenesis in permissive conditions. Overall, these findings further support the capability of platelet-derived factors to activate adipose progenitor cells able to proliferate and differentiate, in agreement with a possible *in vivo* activity of PL on subcutaneous adipose tissue.

In conclusion, we can state that, when a skin injury occurs, the cells of the human subcutaneous adipose tissue exposed to the serum and to the platelet content become activated by the microenvironment changes, modify their secretory profile and exert a regulatory role during the healing process, first promoting an enhancement of the acute inflammatory response, and then favoring the inflammation resolution. We hypothesize that, depending on their particular stage of commitment at the time of injury, adipose progenitor cells either directly re-enter the cell cycle, when still uncommitted, or undergo a de-differentiation process before resuming cell proliferation. This hypothesis is speculative and deserves further investigation. Interestingly, it appears that the less differentiated cells maintain a memory of their initial differentiation status that remains their preferential, if not exclusive, differentiation pathway.

## Author Contributions

AR conception and design, collection and/or assembly of data, data analysis and interpretation, manuscript writing. MM conception and design, data interpretation, manuscript revision. RC conception and design, data interpretation, manuscript writing, financial support. FD conception and design, data analysis and interpretation, manuscript writing.

### Conflict of Interest Statement

The authors declare that the research was conducted in the absence of any commercial or financial relationships that could be construed as a potential conflict of interest.

## Supplementary Material

Supplementary Figure 1 Chondrogenic differentiation of HS-expanded hASC at passage 2 by pellet-culture method. **(A,B)** Representative toluidine blue staining **(A)** and immunohistochemistry for collagen type II **(B)** of paraffin-embedded pellet cross sections (scale bars = 200 μm). The images on the right are magnifications of highlighted areas.
