# Supplementary figures and images for: Platelet Lysate Activates Human Subcutaneous Adipose Tissue Cells by Promoting Cell Proliferation and Their Paracrine Activity Toward Epidermal Keratinocytes
Source: Front Bioeng Biotechnol. 2018 Dec 21;6:203. doi: 10.3389/fbioe.2018.00203 (PMC6308153; doi:10.3389/fbioe.2018.00203)

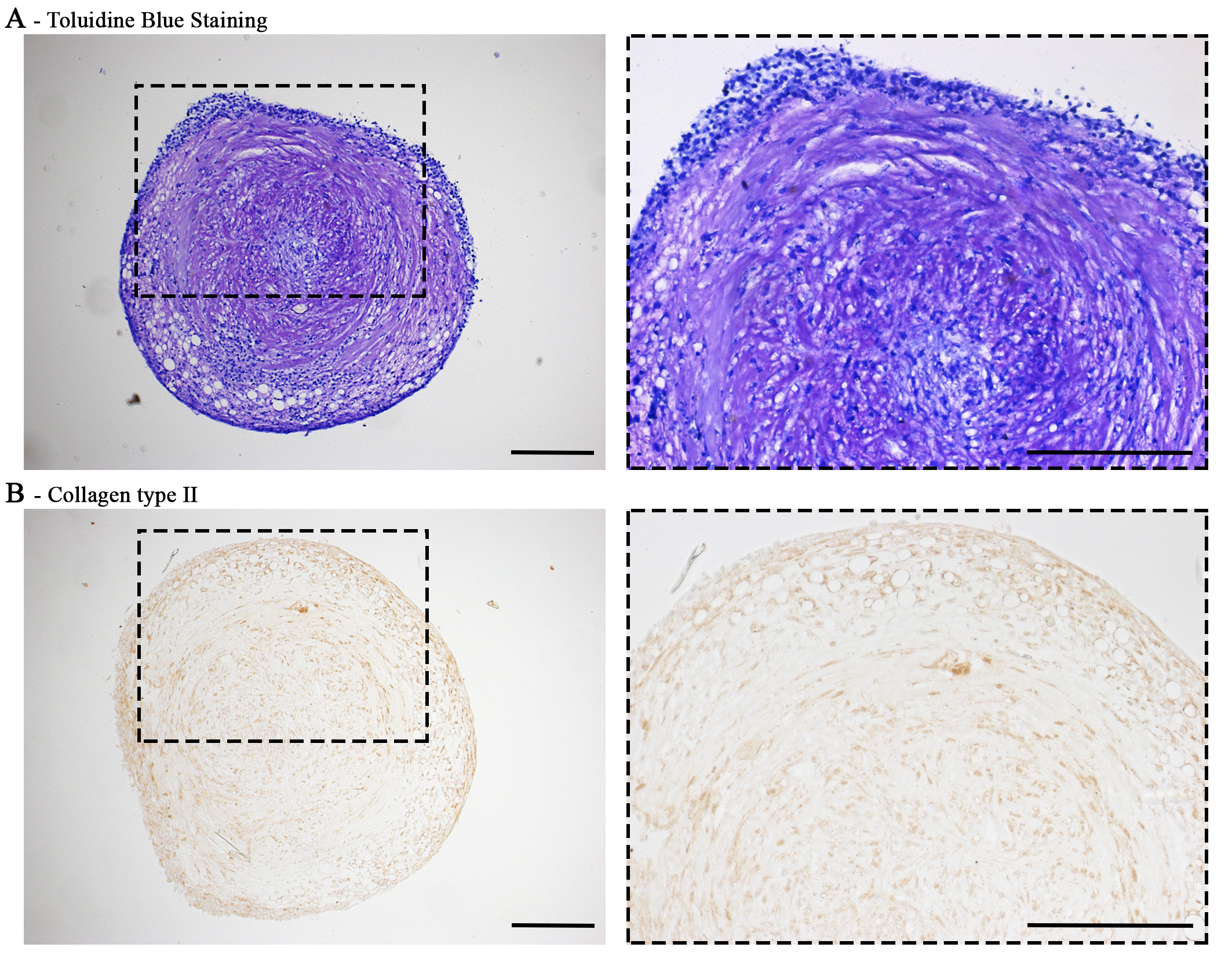

Supplement: Supplementary Figure 1 — Chondrogenic differentiation of HS-expanded hASC at passage 2 by pellet-culture method. (A,B) Representative toluidine blue staining (A) and immunohistochemistry for collagen type II (B) of paraffin-embedded pellet cross sections (scale bars = 200 μm). The images on the right are magnifications of highlighted areas. [file Image_2.TIF]
