# Supplementary material for: Platelet Lysate Activates Human Subcutaneous Adipose Tissue Cells by Promoting Cell Proliferation and Their Paracrine Activity Toward Epidermal Keratinocytes
Source: Front Bioeng Biotechnol. 2018 Dec 21;6:203. doi: 10.3389/fbioe.2018.00203 (PMC6308153; doi:10.3389/fbioe.2018.00203)
Supplement: Supplementary file 2 [file Data_Sheet_1.PDF]

Supplementary Material

Materials

Human apo-transferrin, linoleic acid, bovine serum albumin, sodium pyruvate and hyaluronidase were purchased from Sigma-Aldrich (St. Louis, MO, USA). Recombinant human TGF-β1 was from PeproTech (London, UK). Toluidine blue O salt was obtained from SERVA Electrophoresis GmbH (Heidelberg, Germany). Primary antibody raised against type II collagen was from Developmental Studies Hybridoma Bank (Iowa City, USA).

Chondrogenic differentiation and relative analysis

Chondrogenic differentiation was induced on HS-expanded hASCs at passage 2 by pellet-culture method. After trypsinization, 2.5×10^5 cells in complete medium were put into 15 mL conical tubes and centrifuged at 1500 rpm for 10 min at room temperature to form cell pellets at the bottom of tubes. Tubes were incubated at 37°C in a humidified atmosphere with 5% CO_2 overnight paying attention to do not damage pellets. At the day after, the medium was poured off and, after a washing step with PBS, replaced with serum-free medium supplemented with 6.25 µg/mL insulin, 6.25 µg/mL human apo-transferrin, 1.25 µg/mL linoleic acid, 5.35 µg/mL bovine serum albumin, 1 mM sodium pyruvate, 50 µg/mL ascorbic acid, 10^{-7} M dexamethasone and 10 ng/mL TGF-β1. After 28 induction days, pellets were extensively washed with PBS, fixed with 4% paraformaldehyde for 20 min at 4°C, rinsed extensively with PBS, dehydrated with increasing ethanol concentrations, cleared in xylol and embedded in paraffin. Pellets were sectioned into 5 µm sections and stained. For toluidine blue staining, sections were deparaffinised with xylol, hydrated to water with decreasing ethanol concentrations, stained with 2% toluidine blue O in acetate buffer (pH 5.5) for 35 sec, washed with distilled water, dried off at 37°C for 3 hours, dehydrated to xylol and mounted in Eukitt®. For immunohistochemical investigations, deparaffinised sections were treated following the same indications reported in the paper but with same modifications. After the incubation with H_2O_2, sections were also treated with 1 mg/mL hyaluronidase in PBS (pH 6.0) for 30 min at 37°C to favour primary antibody penetration in the extracellular matrix. The primary antibody raised against type II collagen (1:100 dilution) was added and incubated overnight at 4°C in a humid chamber. The immunodetection was performed using the chromogenic reporter DAB. Images were acquired by microscope Axiovert 200M (Carl Zeiss).
