# Supplementary material for: Platelet Lysate Activates Human Subcutaneous Adipose Tissue Cells by Promoting Cell Proliferation and Their Paracrine Activity Toward Epidermal Keratinocytes
Source: Front Bioeng Biotechnol. 2018 Dec 21;6:203. doi: 10.3389/fbioe.2018.00203 (PMC6308153; doi:10.3389/fbioe.2018.00203)

Figure 3D: full scan of the original blot, used as representative experiment, probed consecutively (on top) with specific primary antibodies.

phospho-ERK$_{1/2}$ (Thr202/Tyr204)

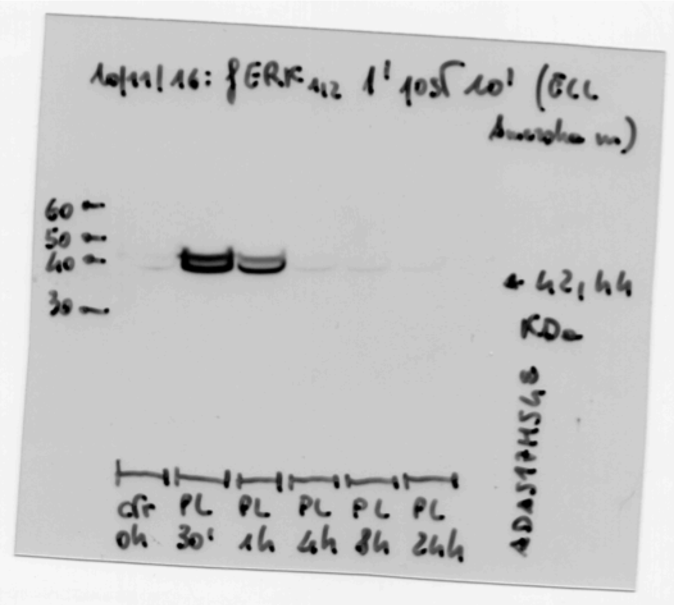

phospho-Akt (Ser473)

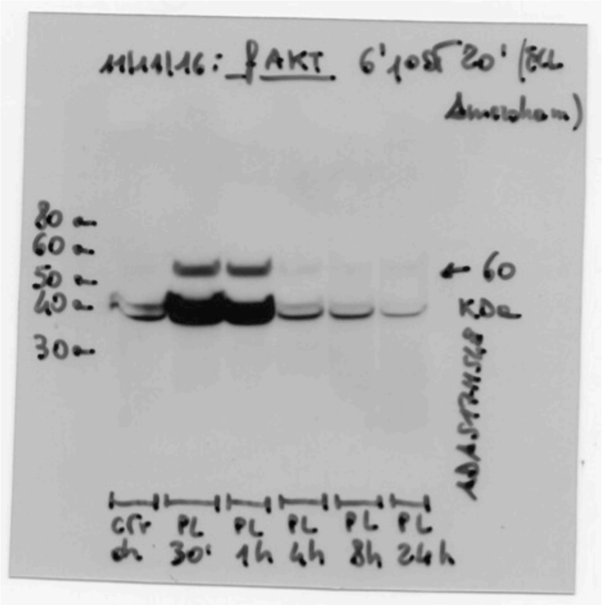

ERK$_{1/2}$

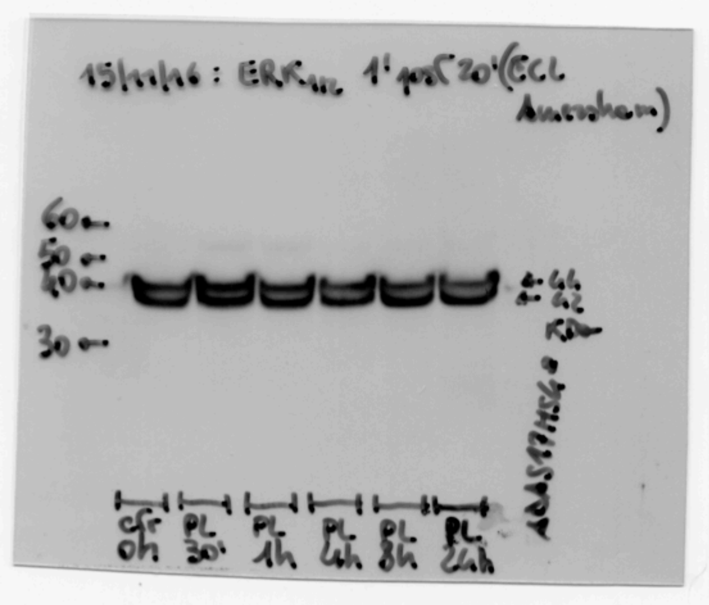

Akt

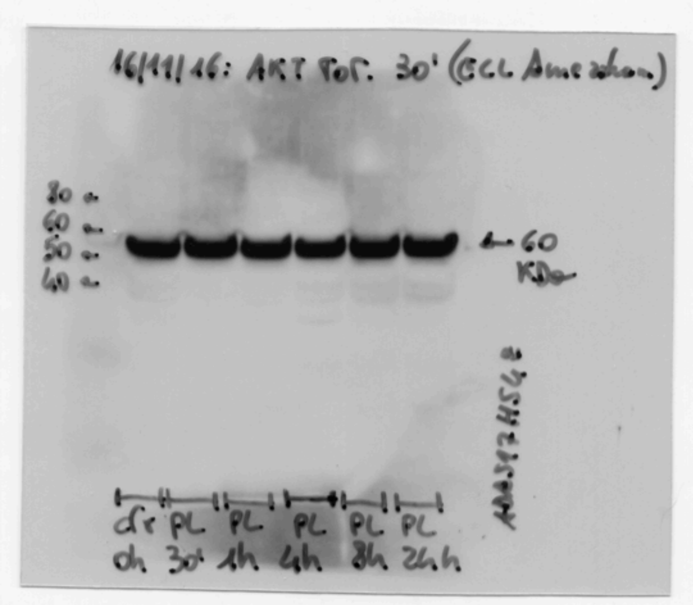

to be continued…

Figure 3D

phospho-STAT3 (Tyr705)

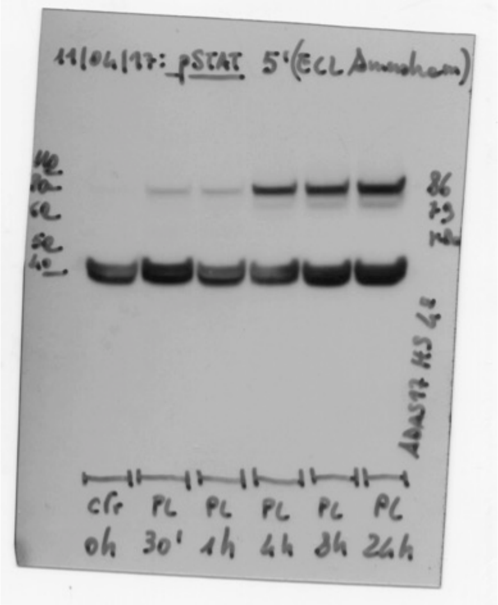

STAT3

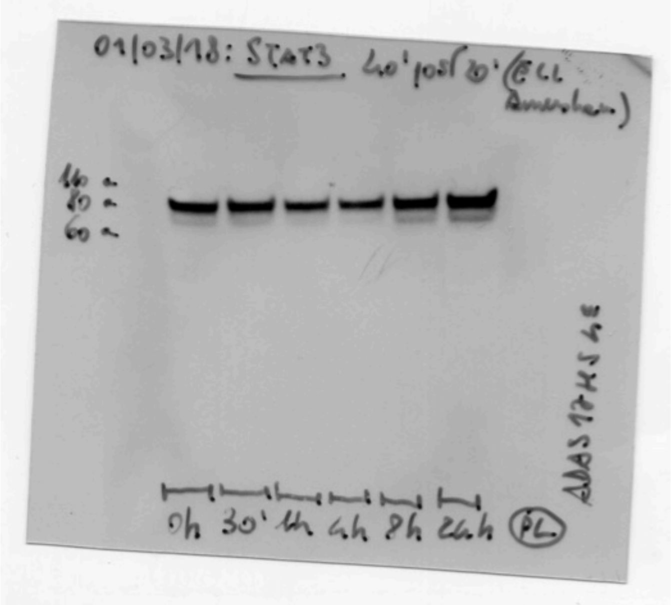

Cyclin D1

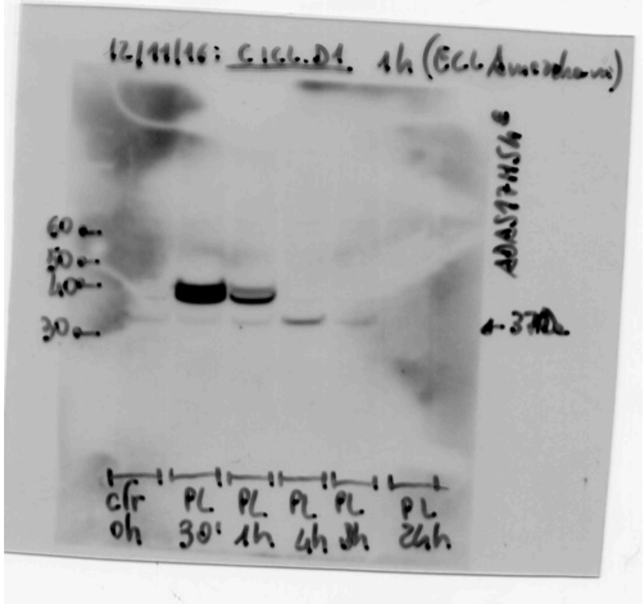

Actin

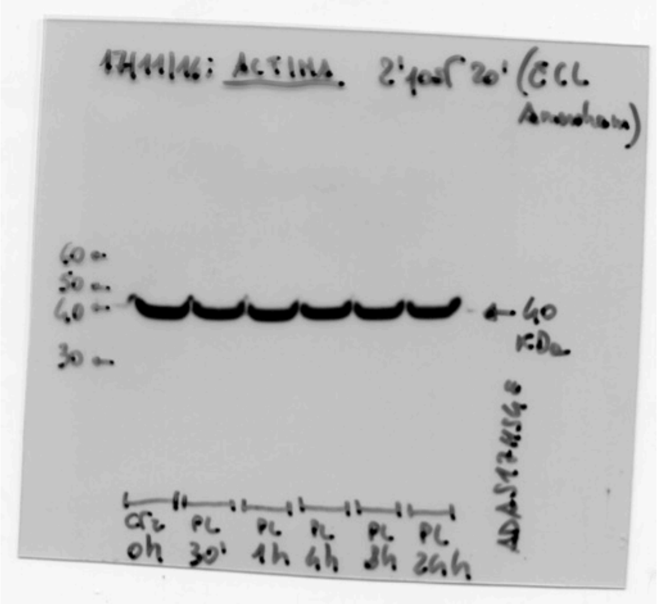

<u>Figure 6C,D</u>: full scan of the original blot, used as representative experiment, probed consecutively (on top) with specific primary antibodies.

IL-6

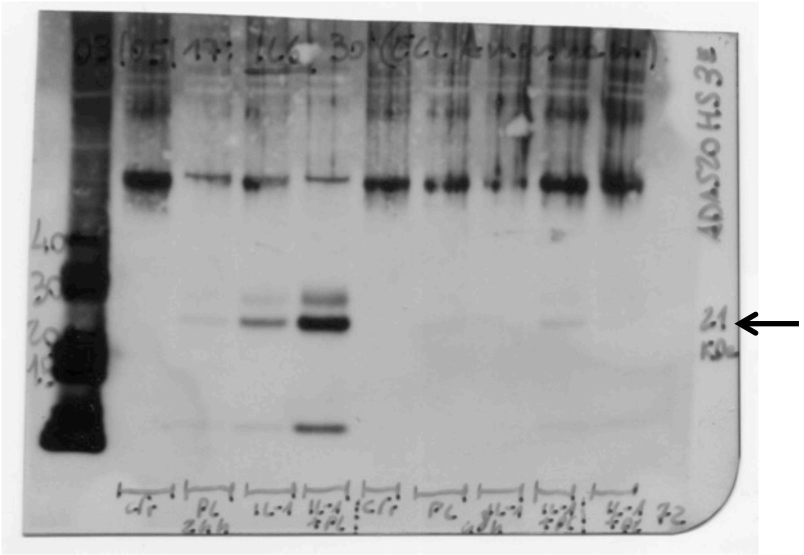

IL-8

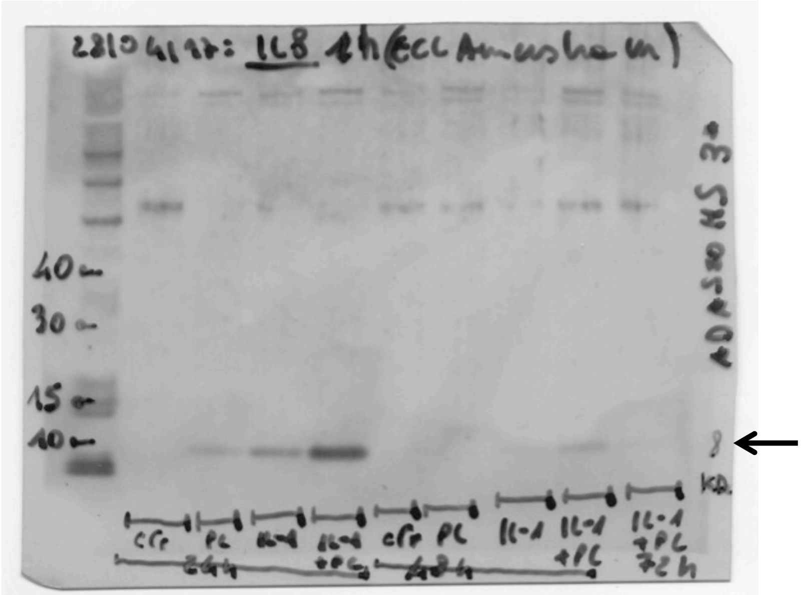

Figure 6E: full scan of the original blot, used as representative experiment, probed consecutively (on top) with specific primary antibodies.

COX-2

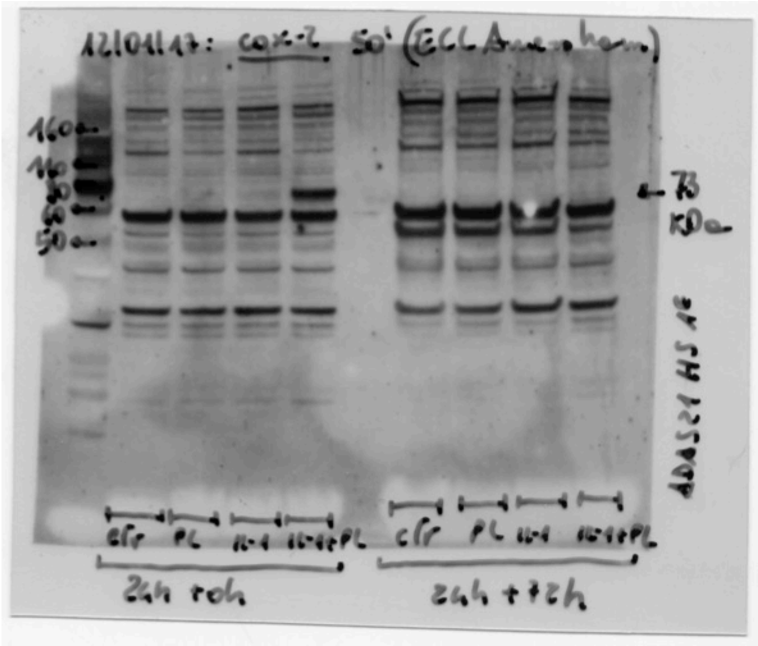

Actin

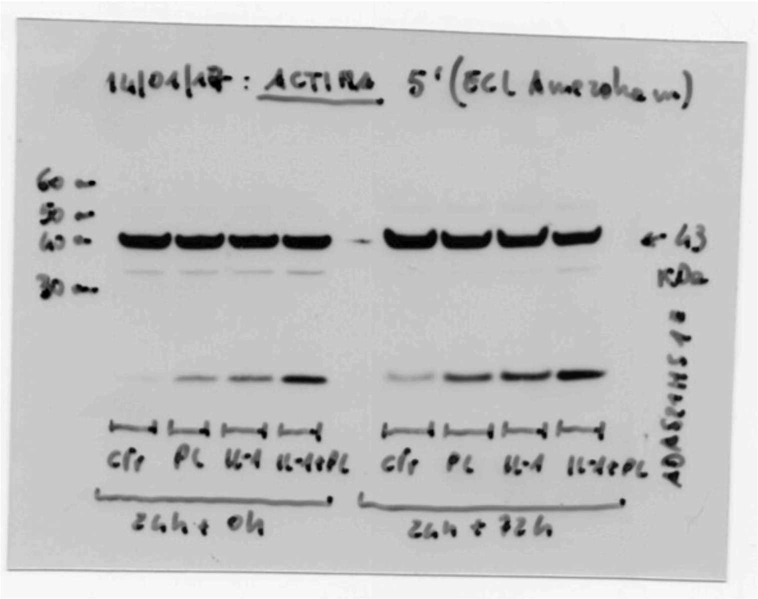

Supplement: Supplementary file 3 [file Data_Sheet_2.PDF]
